# A New Wide Bandgap Donor Polymer for Efficient Nonfullerene Organic Solar Cells with a Large Open‐Circuit Voltage

**DOI:** 10.1002/advs.201901773

**Published:** 2019-08-29

**Authors:** Yumin Tang, Huiliang Sun, Ziang Wu, Yujie Zhang, Guangye Zhang, Mengyao Su, Xin Zhou, Xia Wu, Weipeng Sun, Xianhe Zhang, Bin Liu, Wei Chen, Qiaogan Liao, Han Young Woo, Xugang Guo

**Affiliations:** ^1^ Department of Materials Science and Engineering and The Shenzhen Key Laboratory for Printed Organic Electronics Southern University of Science and Technology (SUSTech) No. 1088, Xueyuan Road Shenzhen Guangdong 518055 China; ^2^ Department of Chemistry Korea University Seoul 136‐713 South Korea; ^3^ eFlexPV Limited (China) Room 228, Block 11, Jin Xiu Da Di, No. 121 Hu Di Pai Song Yuan Sha Community, Guanhu Street, Longhua District Shenzhen Guangdong 518000 China

**Keywords:** complementary absorption, donor polymers, nonfullerene organic solar cells, nonhalogenated solvents, wide bandgap

## Abstract

Significant progress has been made in nonfullerene small molecule acceptors (NF‐SMAs) that leads to a consistent increase of power conversion efficiency (PCE) of nonfullerene organic solar cells (NF‐OSCs). To achieve better compatibility with high‐performance NF‐SMAs, the direction of molecular design for donor polymers is toward wide bandgap (WBG), tailored properties, and preferentially ecofriendly processability for device fabrication. Here, a weak acceptor unit, methyl 2,5‐dibromo‐4‐fluorothiophene‐3‐carboxylate (FE‐T), is synthesized and copolymerized with benzo[1,2‐b:4,5‐b′]dithiophene (BDT) to afford a series of nonhalogenated solvent processable WBG polymers P1‐P3 with a distinct side chain on FE‐T. The incorporation of FE‐T leads to polymers with a deep highest occupied molecular orbital (HOMO) level of −5.60−5.70 eV, a complementary absorption to NF‐SMAs, and a planar molecular conformation. When combined with the narrow bandgap acceptor ITIC‐Th, the solar cell based on P1 with the shortest methyl chain on FE‐T achieves a PCE of 11.39% with a large *V*_oc_ of 1.01 V and a *J*_sc_ of 17.89 mA cm^−2^. Moreover, a PCE of 12.11% is attained for ternary cells based on WBG P1, narrow bandgap PTB7‐Th, and acceptor IEICO‐4F. These results demonstrate that the new FE‐T is a highly promising acceptor unit to construct WBG polymers for efficient NF‐OSCs.

## Graphical abstract

**A series of wide bandgap donor polymers** are designed and synthesized by incorporating a monothiophene functionalized with both a fluorine atom and an ester group. Fabricated from nonhalogenated solvent, power conversion efficiencies of 11.39% and 12.11% are achieved for binary and ternary nonfullerene solar cells, respectively.

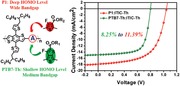

## 1 Introduction

Organic solar cells (OSCs) have been widely studied as an emerging photovoltaic technology with distinctive advantages over traditional solar cells, such as mechanical flexibility, solution‐processability, and compatability with roll‐to‐roll printing.^1^, ^2^, ^3^, ^4^, ^5^, ^6^, ^7^, ^8^, ^9^, ^10^, ^11^ Recently, extensive progresses have been made in nonfullerene organic solar cells (NF‐OSCs). Benefiting from the development of nonfullerene small molecule acceptors (NF‐SMAs), NF‐OSCs have outperformed the state‐of‐the‐art fullerene‐based PSCs and showed power conversion efficiencies (PCEs) over 16%.^12^, ^13^, ^14^, ^15^, ^16^, ^17^ Generally, high‐performance NF‐SMAs possess low‐lying frontier molecular orbital (FMO) energy levels and narrow bandgaps with absorption edges spanning from 700 to 900 nm. To further improve the PCEs of NF‐OSCs, donor polymers with compatible energy levels and absorption spectrum that could complement the SMAs are strongly needed. Hence, it is crucial to develop approaches that could rationally tune the properties of the polymer from the synthetic point of view.

**Figure 1:**
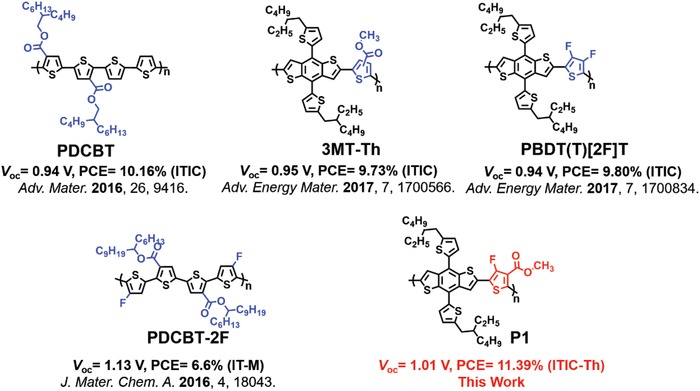
Chemical structures of the representative donor polymers with ester substituted thiophene and fluorine substituted thiophene for NF‐OSCs and the donor polymer P1 in this work. Due to the structural asymmetry, polymer P1 is regiorandom.

Recently, benzo[1,2‐b:4,5‐b′]dithiophene (BDT)‐based D‐A type polymers have been proven to be excellent polymer donor materials for NF‐OSCs.^18^, ^19^, ^20^, ^21^, ^22^, ^23^ For instance, poly[4,8‐bis(5‐(2‐ethylhexyl)thiophen‐2‐yl)benzo[1,2‐b;4,5‐b′]dithiophene‐2,6‐diyl‐alt‐(4‐(2‐ethylhexyl)‐3‐fluorothieno[3,4‐b]thiophene‐)‐2‐carboxylate‐2‐6‐diyl)] (PTB7‐Th, also known as PCE10) has shown numerous impressive achievements in fullerene‐based solar cells.^24^, ^25^, ^26^, ^27^, ^28^ However, due to its relatively high‐lying highest occupied molecular orbital (HOMO) level (around −5.32 eV) and absorption profile that overlaps heavily with NF‐SMAs, it is difficult for PTB7‐Th to realize a high *J*_sc_ and a large *V*_oc_ simultaneously, which significantly limits its applicability in NF‐OSCs. As a result, it is imperative to chemically optimize the building block for wide bandgap (WBG) polymer donors with a deep HOMO level, which should be beneficial for both *V*_oc_ (deep HOMO) and *J*_sc_ (complementary absorption).^29^, ^30^, ^31^, ^32^, ^33^ To this end, an effective strategy is to attach electron‐withdrawing group onto thiophene units. For instance, Hou and coworkers introduced the ester‐substituted thiophene (ET) into the main chain of the polymer, i.e., PDCBT, resulting in a low‐lying HOMO and an enhanced *V*_oc_ (**Figure** **1**).^34^, ^35^ The ET group has also been incorporated into BDT‐based polymer donor 3MT‐Th by Choi and coworkers, resulting in a PCE of 9.73% with a large *V*_oc_ of 0.95 V.^36^ Furthermore, the benefit and success of fluorine‐substituted thiophene (FT) both attached as the side chain and incorporated into the polymer main chain has been demonstrated by various high‐performance NF‐OSCs. For example, as reported by Beaujuge and coworkers, the FT‐*alt*‐BDT polymer named PBDT(T)[2F]T achieved a PCE of 9.8% enabled by an increased *V*_oc_ of 0.94 V and reduced geminate recombination.^37^ To take advantage of the positive effects of both ET and FT, a new polymer semiconductor PDCBT‐2T was synthesized, recently (Figure 1).^38^ However, the increased thiophene content in polymer showed negative effects on the optimization of HOMO levels. In addition, the increased thiophene content could lead to an excessively strong propensity for the polymer to aggregate and thus a poor miscibility with the acceptor molecule.

Apart from the material design, it is also important to choose appropriate solvent and additive to obtain a favorable morphology for the BHJ film.^39^ However, most active layers require halogenated solvents for this purpose, such as chloroform (CF), chlorobenzene (CB), and dichlorobenzene (DCB), which would become issues for industrial scale production and eventually the commercialization of OSC technology. Thus, although it is highly challenging, achieving highly efficient NF‐OSCs by using environmental friendly solvent would have profound impacts in the research field.^6^, ^40^, ^41^, ^42^, ^43^, ^44^

Herein, we designed and synthesized a novel building block methyl 2,5‐dibromo‐4‐fluorothiophene‐3‐carboxylate (FE‐T), a monothiophene functionalized with both fluorine (F) atom and ester (E) group. FE‐T was copolymerized with BDT to afford a new polymer donor P1 (Figure 1). As expected, the synergetic effects of both electron‐withdrawing F and ester groups could result in downshifted energy levels of the BDT‐based polymer, the major advantage enabled by the new FE‐T. As a result, the P1‐based NF‐OSCs yielded a large *V*_oc_ (>1.0 eV) with a small low energy loss of ≈0.57 eV. Furthermore, we varied the side chain on FE‐T and found that the shortest chain (of P1) enabled the most favorable morphology in the BHJ film and thus the best device performance. Compared with the benchmark donor polymer PTB7‐Th where the fluorine atom and ester group were attached on thieno[3,4‐b]‐thiophene, P1 exhibited a deeper HOMO level and a wider bandgap (1.95 eV). Solar cell devices with P1:ITIC‐Th active layer were fabricated with a halogen‐free solvent (toluene), which exhibited a higher *V*_oc_ and a larger PCE than PTB7‐Th:ITIC‐Th–based cells. The PCE (11.39%) achieved by the P1‐based devices was among the top values reported for binary NF‐OSCs using a halogen‐free solvent. Finally, by employing a ternary approach, the PCE was further improved to over 12%. These results show the great success of our design principle and demonstrate that the FE‐T unit is a promising electron‐withdrawing building block to develop wide bandgap donor polymers for efficient NF‐OSCs.

## 2 Results and Discussion

Three WBG polymers P1, P2, and P3 with distinct side chain on the FE‐T unit were synthesized by Stille coupling‐based polycondensation reaction of two monomers, i.e., BDT and FE‐T, and their molecular structures are shown in Figure 1a. Synthetic details of the dibrominated FE‐T monomers and the polymers P1–P3 can be found in the Supporting Information. The synthesis of the FE‐T monomer precursors was similar to the approach reported in our previous works,^45^, ^46^, ^47^ followed by bromination and transesterification to afford the dibrominated FE‐T monomers with an overall yield >40% (Scheme S1, Supporting Information). Notably, this series of polymers were favorably dissolved not only in normal halogenated solvents such as chloroform (CF), chlorobenzene (CB), and dichlorobenzene (DCB), but also in nonhalogenated solvents like toluene, *o*‐xylene and trimethylbenzene (TMB), which was similar to PTB7‐Th.^36^ Molecular weights of the three polymers were measured by high‐temperature gel permeation chromatography (GPC). The number‐average molecular weights (*M*_n_s) of polymers were ≈30 kDa, and polydispersity indexes (PDIs) were around 2.0 (**Table** **1**). Besides, decent thermal stability with a decomposition temperature (*T*_d_) above 320 °C was observed in the thermogravimetric analysis (TGA) (Figure S1, Supporting Information) for the polymers.

**Table 1:** Molecular and optoelectronic properties of polymers P1, P2, and P3

Polymer	M_n_ [KDa]	PDI	λ_onset,film_ [nm]	λ_peak,film_ [nm]	λ_onset,solution_ [nm]	λ_peak,solution_ [nm]	*E*_HOMO_ ^a)^	*E*_LUMO_ ^b)^	*E*_g_^opt^ ^c)^
P1	32.2	2.1	637	540 570	602	502 565	5.60	3.65	1.95
P2	38.2	2.2	614	525 560	567	493	5.70	3.68	2.02
P3	43.9	2.0	617	530 565	563	491	5.71	3.70	2.01

^a)^ Calculated from cyclic voltammetry (CV) method

^b)^ Derived from *E*_HOMO_ and *E*_g_^opt^

^c)^ Calculated from *E*_g_^opt^ = 1240/λ_onset_.

**Figure 2:**
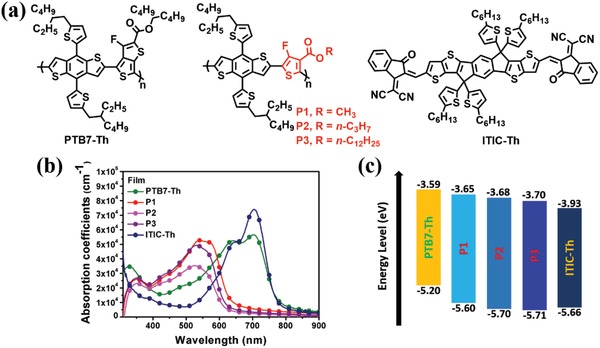
a) Chemical structures of donor polymers and nonfullerene small molecule acceptor in this work. Due to the structural asymmetry, polymers P1‐P3 are regiorandom; b) absorption coefficient spectra of PTB7‐Th, P1‐P3, and ITIC‐Th in film state; c) energy level alignment for PTB7‐Th, P1‐P3, and ITIC‐Th.

It is clear that P1, P2, and P3 exhibit absorption spectrum more complementary to ITIC‐Th than PTB7‐Th. As shown in **Figure** **2**b, compared to PTB7‐Th that exhibited an absorption peak at 700 nm, P1, P2, and P3 showed hypsochromic shifts and the absorption peaks at around 560–570 nm were observed. This could be ascribed to decreased quinoidal population in polymers P1–P3, which was consistent with the density functional theory (DFT) calculation shown in Figure S11 in the Supporting Information. Furthermore, side‐chain engineering revealed that the absorption coefficient and π–π stacking were weakened (Figure 2b) as the alkyl chain length was increased from CH_3_ (P1) to *n*‐C_3_H_7_ (P2) or *n*‐C_12_H_25_ (P3). Different from PTB7‐Th that exhibited relatively strong aggregation in both solution and film states, P1 showed weak aggregation in solution (concentration: 0.01 mg mL^−1^) but strong stacking in film state, evidenced by its absorption spectra (Figure S12, Supporting Information and Figure 2b). This property can be used to effectively tune the morphology of the BHJ active layer during device fabrication. In addition, it was found that the absorption coefficient of P1 was comparable to that of PTB7‐Th, as depicted in Figure 2b.

The cyclic voltammetry (CV) curves (Figure 2c) revealed that the HOMO of P1 was at −5.60 eV, much deeper than that of PTB7‐Th (−5.20 eV), and the lowest unoccupied molecular orbit (LUMO) was −3.65 eV for P1 that was also lower than that of PTB7‐Th (−3.59 eV), as summarized in Table 1. From these results, P1 has a wider bandgap (1.95 eV) than PTB7‐Th (1.57 eV), and the lower‐lying P1 HOMO could increase the *V*_oc_ for the P1‐based NF‐OSCs. In addition, benefiting from both the high electronegativity of F atom and the strong electron‐withdrawing capability of ester group, incorporating FE‐T into polymer leads to the resulting semiconductor with lower‐lying FMO energy levels than the polymer analogues with either F atom or ester functional group.^36^, ^37^ Notably, Figure S14 in the Supporting Information and Table 1 show that the HOMO level could be further deepened by the extending the side‐chain in the ester group, e.g., −5.70 and −5.71 eV for P2 and P3, respectively.

To evaluate the photovoltaic performance of the devices fabricated from both halogenated and nonhalogenated solvents, NF‐OSCs were constructed with a conventional device configuration of glass/ITO/PEDOT:PSS/active layer/Ca or PFN‐Br/Al and characterized under AM1.5G solar illumination (100 mW cm^−2^). To improve the device performance, systematic optimizations were performed involving donor/acceptor ratios, processing solvents, solution concentrations, thermal annealing, solvent additives, and interfacial layers, and the details of these experiments are shown in **Table** **2** and Supporting Information. It is clear that the device performance of solar cells processed using the halogenated solvent CB are better than those using nonhalogenated solvents. But the PCE difference is quite small (less than 1.0%), which indicates that nonhalogenated solvents can also enable high‐performance devices for our polymers.

**Table 2:** Device performance parameters of nonfullerene organic solar cells based on PTB7‐Th:ITIC‐Th and P1:ITIC‐Th active layer

Materials	Solvent	Thickness [nm]	PCE_max_ */*(PCE*_avg_*) [%]	*V*_oc_ [V]	*J*_sc_ ^a)^/(*J*_sc_^avg^) [mA cm^−2^]	*J*_sc_ ^b)^ [mA cm^−2^]	FF [%]	*E*_g_^onset^ ^c)^ [eV]	*E*_loss_ [eV]
PTB7‐Th:ITIC‐Th	Toluene	120	8.25/(8.16 ± 0.09)	0.80	14.85/(14.59 ± 0.26)	14.81	69.46	1.56	0.76
P1:ITIC‐Th	Toluene	130	11.39/(11.17 ± 0.22)	1.01	17.89/(17.66 ± 0.23)	17.83	63.05	1.58	0.57

^a)^ From *J*–*V* measurements

^b)^ Integrated from EQE

^c)^ Calculated from EQE spectrum. The numbers in the parentheses are the average values with standard deviations for 10 solar cell devices.

**Figure 3:**
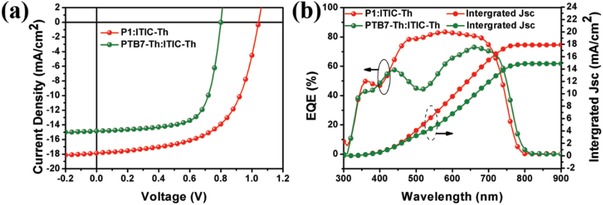
a) The *J*–*V* curves of the nonfullerene organic solar cells based on PTB7‐Th:ITIC‐Th and P1:ITIC‐Th active layer; b) the EQE spectra of the corresponding solar cells.

Shown in **Figure** **3**a are the *J*–*V* characteristics of the optimized NF‐OSCs. Notably, the *V*_oc_ of device based on P1 was 1.01 V, which was significantly higher than that of PTB7‐Th (0.80 V), consistent with the deeper‐lying HOMO level of P1. Compared to PTB7‐Th that showed a *J*_sc_ of 14.85 mA cm^−2^_,_ the *J*_sc_ of P1‐based device is much higher (17.89 mA cm^−2^), which could be attributed to the enhanced carrier mobility, decreased leakage current and suppressed charge recombination (vide infra). Thus, with fill factors of 63.05% and 69.46% for P1 and PTB7‐Th‐based NF‐OSCs, respectively, the PCE of P1‐based NF‐OSCs reached 11.39%, showing a substantial increase compared to that of PTB7‐Th‐based NF‐OSCs (8.25%). The high PCE of P1‐based NF‐OSCs is comparable to the best values in literatures where ITIC‐Th was used as the only acceptor, as shown in Table S13 in the Supporting Information. Particularly, considering that the processing solvent was halogen‐free, the performance of P1‐based devices is remarkable. To further evaluate the performances of P1‐based devices, other NF‐SMAs are also used to fabricate NF‐OSCs, including the state‐of‐the‐art Y6,^13^ and the detailed device performance parameters under the optimized condition are included in Table S2 in the Supporting Information. The P1:Y6 based cells yield a higher *J*_sc_ (20.57 mA cm^−2^) than the P1:ITIC‐Th based ones (17.89 mA cm^−2^), which is attributed to the improved absorption of Y6 in infrared region. However, it showed a lower PCE of 10.87% due to the reduced *V*_oc_ and FF. It was found that the LUMO offset between the donor material and the acceptor material is one of the major contributors to energy loss (*E*_loss_) in OSCs. In comparison to ITIC‐Th, Y6 showed a lower‐lying LUMO level, which hence resulted in the smaller *V*_oc_ in P1:Y6–based solar cells.

External quantum efficiencies (EQEs) for PTB7‐Th and P1‐based devices were measured and the results are shown in Figure 3b and Table 2. The EQE of the device based on P1:ITIC‐Th is quite high in the range of 300–720 nm, and the peak at around 500 nm is mainly due to the absorption of P1 (Figure S13, Supporting Information). Integrated *J*_sc_s from EQE are 17.83 mA cm^−2^ for P1‐based device and 14.81 mA cm^−2^ for PTB7‐Th‐based device, which are well consistent with the values from the *J*–*V* characteristics. The *E*_loss_ was calculated based on the equation: *E*_loss_ = *E*_g_^onset^−e*V*_oc_, where *E*_g_^onset^ was obtained from the curve of EQE. As summarized in Table 2, the *E*_loss_ of P1‐based device was 0.57 eV that was greatly smaller than that of PTB7‐Th‐based cell (0.76 eV).

Additionally, the side‐chain effect of FE‐T was investigated by fabricating devices with polymers P2 and P3 and the same acceptor ITIC‐Th, and the results are shown in Figure S15 and Table S10 in the Supporting Information. As the side‐chain length increases (R = *n*‐C_3_H_7_ for P2 and *n*‐C_12_H_25_ for P3), the PCE first decreases to about 2.35% for P2:ITIC‐Th with a *V*_oc_ of 0.91 V, a *J*_sc_ of 8.68 mA cm^−2^ and a FF of 29.66%, and then increases for P3:ITIC‐Th to 3.35% with a *V*_oc_ of 1.00 V, a *J*_sc_ of 9.62 mA cm^−2^, and a FF of 34.65%. Figure S16 in the Supporting Information shows the EQE of the devices in which below 50% of EQE for P2:ITIC‐Th and P3:ITIC‐Th are observed. Besides the unmatched energy levels between P2 (or P3) and the acceptor, the inferior device performances indicate undesirable morphology in the blend films based on polymers with longer alkyl chains, which could interrupt the molecular packing for the donor and acceptor. To better understand the effect of alkyl side‐chain, which is the main chemical variable among P1, P2, and P3, atomic force microscopy (AFM), transmission electron microscopy (TEM), and grazing‐incidence wide‐angle X‐ray scattering (GIWAXS) experiments were carried out with the results shown in Figures S19–21 in the Supporting Information. From the AFM results, it was found that longer side‐chain slightly increases the root‐mean‐square (RMS) roughness to 0.71 and 0.86 nm for P2:ITIC‐Th and P3:ITIC‐Th blend, respectively. From TEM results and the weaker diffraction peaks in the GIWAXS results, it can be concluded that the molecular order is significantly disrupted in both the pure and blend films based on P2 and P3, particularly in the out‐of‐plane direction (less face‐on), not only for the polymer, but also for ITIC‐Th as well. The increased length of alkyl side‐chain in P2 and P3 could facilitate more entanglement and less orientation after blended with ITIC‐Th, along with the weaker π–π stacking, are the main reasons for the reduced device performance when compared to P1.

**Figure 4:**
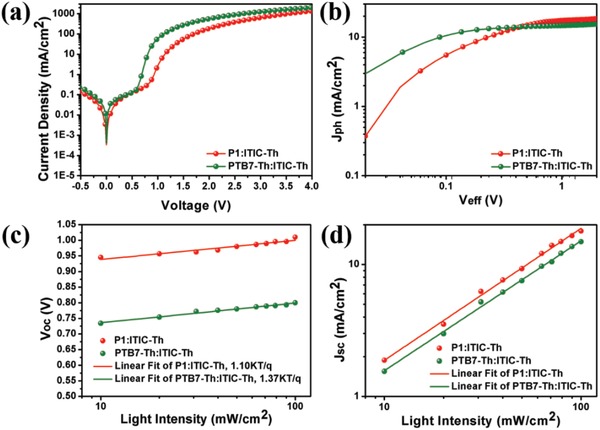
a) Dark *J*–*V* curves, b) *J*_ph_–*V*_eff_ characteristics, c) *V*_oc_ versus light intensity and d) *J*_sc_ versus light intensity of optimized nonfullerene organic solar cells containing PTB7‐Th:ITIC‐Th and P1:ITIC‐Th active layer.

For more consistent comparison between PTB7‐Th and P1, photoluminescence (PL) measurements were first performed on the pure films of both polymers together with ITIC‐Th as well as the PTB7‐Th:ITIC‐Th and P1:ITIC‐Th blend films (Figure S23, Supporting Information). The neat films of PTB7‐Th, P1, and ITIC‐Th showed emission peaks at 760, 654, and 761 nm, respectively. When blended with ITIC‐Th, the PL quenching efficiencies of PTB7‐Th:ITIC‐Th and P1:ITIC‐Th were calculated to be 75.4% and 99.6%, respectively, in good accord with the trend of their *J*_sc_s. Shown in **Figure** **4**a are the dark *J*–*V* characteristics for the PTB7‐Th:ITIC‐Th and P1:ITIC‐Th blend films, the P1:ITIC‐Th blend exhibited a lower leakage current under reverse bias than the PTB7‐Th:ITIC‐Th film, indicating a higher shunt resistance of P1:ITIC‐Th blend. Figure 4b depicts the photocurrent density (*J*_ph_) against effective voltage (*V*_eff_) for PTB7‐Th:ITIC‐Th and P1:ITIC‐Th. The photocurrent density *J*_ph_ is defined as *J*_L_ − *J*_D_, where *J*_L_ and *J*_D_ are the current densities under light illumination and in the dark, respectively. *V*_eff_ is given as *V*_0_ − *V*_app_, where *V*_0_ is the compensation voltage when *J*_ph_ = 0 V, and *V*_app_ is the applied voltage. *J*_ph_s saturated at a *V*_eff_≈ 2.0 V for both NF‐OSCs based on PTB7‐Th:ITIC‐Th and P1:ITIC‐Th, which indicated almost all photo‐generated charge carriers were collected when *V*_eff_ > 2.0 V. The exciton dissociation probability (*P*(*E,T*)) was probed, which is defined as *J*_ph_/*J*_sat_, where *J*_sat_ is the saturated current density. The *P*(*E,T*)s is 98% for P1:ITIC‐Th and 97% for PTB7‐Th:ITIC‐Th, suggesting sufficient charge separation in both optimized devices.

Furthermore, the *J*–*V* curves as a function of illumination intensity were measured to investigate the charge recombination. Firstly, the function of light intensity (*I*) on *V*_oc_ was studied for devices of PTB7‐Th:ITIC‐Th and P1:ITIC‐Th, and the curves are shown in Figure 4c. The *V*_oc_ was proportional to *S*ln(*I*) in principle, where the *S* is the slope of the linear fit derived from the semi‐logarithmic curve and could be formatted as *S ∝ nkT*/*q*, where *n*, *k*, *T*, and *q* are the ideality factor, the Boltzmann constant, Kelvin temperature, and elementary charge, respectively. Basically, when the value of *S* is 1*kT*/*q*, the device can be described by the drift‐diffusion model with quasi‐Fermi levels aligned throughout the device with trap‐free recombination behavior. P1:ITIC‐Th device exhibits less trap‐assisted recombination than PTB7‐Th:ITIC‐Th cell, as the values of *S* are 1.10 and 1.37 for P1:ITIC‐Th and PTB7‐Th:ITIC‐Th devices, respectively. Furthermore, Figure 4d shows the dependence of *J*_sc_ on light intensity, which could be described by *J*_sc_ *∝ I^α^*. Under short‐circuit condition, if the bimolecular recombination is negligible, α is unity, while α values of 0.99 and 0.98 were obtained for the devices based on P1:ITIC‐Th and PTB7‐Th:ITIC‐Th, respectively, revealing that bimolecular recombination was not significant in both devices.

The mobilities of the blend films of PTB7‐Th:ITIC‐Th and P1:ITIC‐Th were probed by the space charge limited current (SCLC) method and the results are shown in Figure S22 and Table S12 in the Supporting Information. The hole‐only device and electron‐only device were fabricated with a structure of ITO/PEDOT:PSS/active layer/MoO_3_/Ag and ITO/ZnO/active layer/PFN‐Br/Al, respectively. The hole mobilities are 1.8 × 10^−4^ and 4.8 × 10^−4^ cm^2^ V^−1^ s^−1^ for PTB7‐Th:ITIC‐Th and P1:ITIC‐Th blends, respectively, and the electron mobilities for PTB7‐Th:ITIC‐Th and P1:ITIC‐Th films are 2.0 × 10^−4^ and 3.2 × 10^−4^ cm^2^ V^−1^ s^−1^, respectively, indicating that the carrier transport was greatly improved for the devices based on the P1:ITIC‐Th blend film, which is consistent with its better photovoltaic performance. Notably, the *µ*_e_/*µ*_h_ values are 1.11 and 0.67 for PTB7‐Th:ITIC‐Th and P1:ITIC‐Th films, respectively. The less balanced electron and hole mobility for P1:ITIC‐Th could be a reason for its lower FF, which also suggests the direction for enhancing device performance in the future.

**Figure 5:**
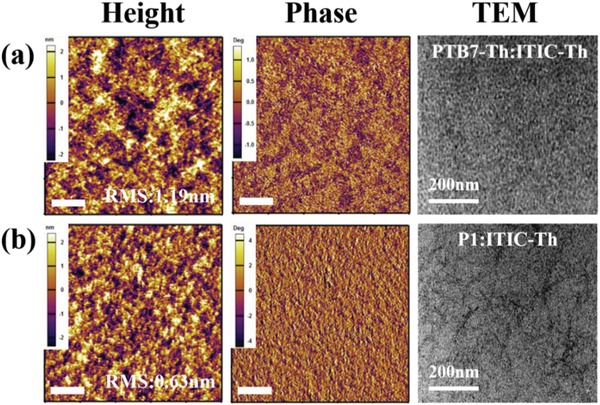
AFM height, phase images, and TEM images of a) PTB7‐Th:ITIC‐Th blend film and b) P1:ITIC‐Th blend film; Scale bar in AFM images: 1.0 um.

To correlate the device performance with film morphology, atomic force microscopy and transmission electron microscopy measurements were carried out with the results shown in **Figure** **5**. The root‐mean‐square surface roughness is 0.63 nm for P1:ITIC‐Th which is smaller than that for PTB7‐Th:ITIC‐Th (1.19 nm) prepared with solvent benzene. Both roughnesses are quite small, but P1:ITIC‐Th film shows a smoother surface than PTB7‐Th:ITIC‐Th blend. The AFM results exhibit good phase separation between the donor and acceptor for both active layers. In the TEM images, domain sizes of around 20 nm with bicontinuous interpenetrating networks were clearly observed for both PTB7‐Th:ITIC‐Th and P1:ITIC‐Th blend films. Slightly larger domains can be observed in the TEM image of P1:ITIC‐Th film, which could be the larger aggregates/crystallites due to its stronger aggregation (Figure 2b). In addition, AFM and TEM characterizations were also employed to further probe the morphology of both blend films processed from chlorobenzene (CB). As shown by Figure S18 in the Supporting Information, both blend films exhibit uniform surface morphologies with RMS roughness of 2.78 and 1.94 nm for PTB7‐Th:ITIC‐Th and P1:ITIC‐Th, respectively. These values are slightly higher compared to that of film processed from toluene, which is likely attributed to the larger aggregates in the blend films enabled by CB processing. Furthermore, the domain sizes of the blends become larger as revealed by the TEM images (Figure S18, Supporting Information). Well‐defined nanoscale interpenetrating networks are maintained in both blends, which are beneficial for exciton dissociation and charge carrier transport.

**Figure 6:**
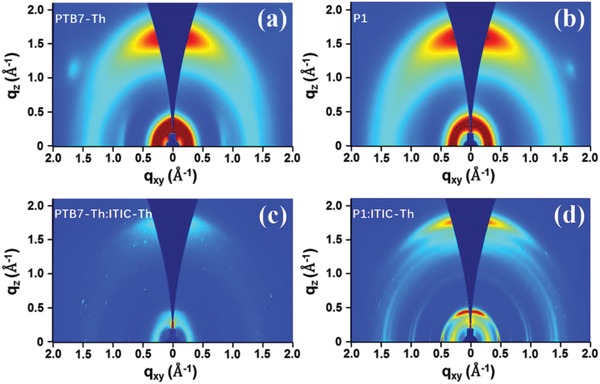
2D GIWAXS images of neat films based on a) PTB7‐Th and b) P1, and blend films of c) PTB7‐Th:ITIC‐Th and d) P1:ITIC‐Th.

To gain deeper insights into the morphology, 2D grazing‐incidence wide‐angle X‐ray scattering was performed for neat films of PTB7‐Th, P1, and ITIC‐Th as well as the PTB7‐Th:ITIC‐Th and P1:ITIC‐Th blend films, with the scattering patterns shown in **Figure** **6** and Figure S17 in the Supporting Information. It is clear that both neat films and blend films exhibit a bimodal orientation with preferential face‐on orientation. Furthermore, strong and sharp peaks in both in‐plane and out‐of‐plane directions can be observed in P1:ITIC‐Th blend film while only weak, less sharp peaks are shown in PTB7‐Th:ITIC‐Th, indicating a more amorphous nature of the film caused possibly by the better miscibility between PTB7‐Th and ITIC‐Th, consistent with its lower electron and hole mobilities. As shown in Table S11 in the Supporting Information, the P1:ITIC‐Th blend film shows a coherence length of 5.71 nm for the π–π stacking, which is much greater than that of PTB7‐Th:ITIC‐Th (1.46 nm) blend film. This agrees with the results of DFT calculation (Figure S11, Supporting Information) and the TEM, which all suggests a stronger crystallization/aggregation tendency of P1. The more ordered domains are beneficial for the charge transport and *J*_sc_.

**Figure 7:**
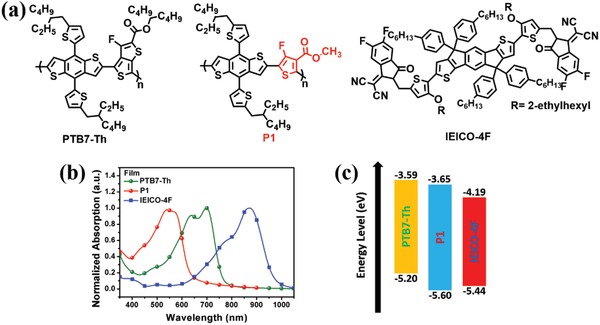
a) Chemical structures of PTB7‐Th, P1 and acceptor IEICO‐4F used in the ternary nonfullerene organic solar cells; b) normalized absorption spectra of PTB7‐Th, P1, and IEICO‐4F in film state; c) energy level alignment of PTB7‐Th, P1, and IEICO‐4F.

**Figure 8:**
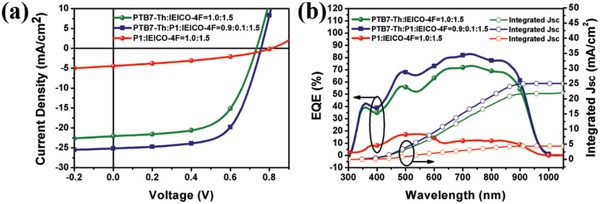
The a) *J*–*V* curves and b) EQE spectra of the optimized ternary nonfullerene organic solar cells based on PTB7‐Th:P1:IEICO‐4F active layer fabricated from toluene solution under illumination (simulated AM 1.5G) at 100 mW cm^−2^.

Since the absorption spectra of PTB7‐Th and P1 are complementary to each other, it would be interesting to fabricate ternary solar cells to take advantage of the NBG and WBG polymers (PTB7‐Th and P1).^48^ To this end, another SMA IEICO‐4F was chosen as the small molecule acceptor because it had the highly complementary absorption to the two polymer donors. The chemical structures of the materials used to fabricate the ternary NF‐OSC are shown in **Figure** **7**a. The absorption edge of IEICO‐4F is about 1000 nm, and the corresponding optical band gap (*E*_g_) is 1.25 eV.^49^ Therefore, the ternary blend films cover a broad spectrum range from 300 to 1000 nm, which is a desirable feature for enhancing light harvesting and thus increasing photocurrent generation in NF‐OSCs. Toluene was still used as the solvent to ensure a halogen‐free fabrication process. The PCE of the optimized ternary NF‐OSC based on PTB7‐Th(0.9):P1(0.1):IEICO‐4F(1.5) reached 12.11%, which showed a clear improvement over the 9.79% PCE of the binary NF‐OSC fabricated with PTB7‐Th(1.0):IEICO‐4F(1.5) active layer. Considering that the PCE of the solar cells with P1(1.0):IEICO‐4F(1.5) active layer was only 1.31%, the performance of the ternary device was remarkable. Although the binary device of P1 and IEICO‐4F did not achieve high performance, there have also been reports that the device performance could be enhanced despite that the third component showed low performance in its own binary device, which reflects the complexity of the morphology and working mechanism of ternary OSCs.^50^, ^51^ The high ternary device performance could mainly be attributed to the similar chemical structure but largely different properties between PTB7‐Th and P1. Other than their complementary absorption, their energy levels are also well matched (Figure 7b,c), which results in the high *J*_sc_ of 25.18 mA cm^−2^ (**Figure** **8**ab and **Table** **3**). The high *J*_sc_ was verified by the EQE of the ternary NF‐OSC where the responses were higher than the corresponding binary cell, particularly in the range of 450–650 nm due to the P1 incorporation.

**Table 3:** Photovoltaic parameters of nonfullerene organic solar cells based on PTB7‐Th:P1:IEICO‐4F

Materials	Solvent	Thickness [nm]	PCE*_max_/*(PCE_avg_) [%]	*V*_oc_ [V]	*J*_sc_ ^a)^/(*J*_sc_^avg^) [mA cm^−2^]	*J*_sc_ ^b)^ [mA cm^−2^]	FF [%]
PTB7‐Th(1.0):IEICO‐4F(1.5)^c)^	Toluene	110	9.79/(9.63 ± 0.16)	0.73	22.06/(21.92 ± 0.14)	22.01	60.47
PTB7‐Th(0.9):P1(0.1):IEICO‐4F(1.5)^c)^	Toluene	130	12.11/(12.02 ± 0.09)	0.74	25.11/(24.98 ± 0.13)	24.89	65.18
P1(1.0): IEICO‐4F(1.5)^c)^	Toluene	120	1.31/(1.09 ± 0.22)	0.81	4.43/(4.20 ± 0.23)	4.36	36.70

^a)^ From *J*–*V* measurements

^b)^ Integrated from EQE

^c)^ The values in parentheses meant mass ratios. The numbers in the parentheses are the average values with standard deviations for 10 solar cell devices.

With the weight ratio of P1 in PTB7‐Th:P1:IEICO‐4F increases to 20%, 30%, and further to 40%, the ternary blends showed higher absorption in the short wavelength region, as displayed in Figure S24 in the Supporting Information. However, increasing the P1 fraction beyond 10% yielded lower device performance. Importantly, compared to the data in literatures (Table S14, Supporting Information), the 12.11% PCE of the ternary device was among the highest values for OCSs processed from relatively green solvents.

To understand the mechanism of the ternary devices, PL measurements were carried out and the results are shown in Figure S26 in the Supporting Information.^52^, ^53^, ^54^ First, the neat films of PTB7‐Th, P1 and IEICO‐4F were characterized and the emission peaks were found at 760, 654, and 950 nm, respectively. Next, binary blend films formed by either two components out of PTB7‐Th, P1, and IEICO‐4F were prepared. For PTB7‐Th:IEICO‐4F and P1:IEICO‐4F, the emission peaks of PTB7‐Th and P1 are quenched by 95.9% and 99.7% by IEICO‐4F, respectively, and those of IEICO‐4F are quenched by 93.2% and 96.5% by PTB7‐Th and P1, respectively, indicating efficient electron (or hole) transfer from donors (or acceptor) to acceptor (or donor). Furthermore, in the blend film of PTB7‐Th:P1, the emission of P1 is significantly quenched by PTB7‐Th, while the emission PTB7‐Th increases, indicating energy transfer between PTB7‐Th and P1.^55^, ^56^, ^57^ Another evidence for the possible energy transfer is that the absorption of PTB7‐Th and the emission of P1 largely overlap. Finally, the ternary blend film of the optimized device that based on PTB7‐Th:P1:IEICO‐4F active layer was characterized through PL measurement, and the emission peak of PTB7‐Th, P1 and IEICO‐4F are quenched by 95.5%, 98.7%, and 94.1%, respectively.

## 3 Conclusion

In summary, we report the design, synthesis and application of a series of new wide bandgap donor polymers P1–P3 based on a novel electron‐withdrawing acceptor unit named FE‐T, which was obtained by introducing both ester and fluorine functional groups on the same single thiophene. Enabled by the improved complementary absorption, deeper‐lying HOMO level and favorable morphology in blend film, the best‐performing polymer P1 with the shortest methyl group on FE‐T exhibited a high PCE of 11.39% with a *J*_sc_ of 17.89 mA cm^−2^, a *V*_oc_ of 1.01 V, and a FF of 63.05%. Notably, the performance was realized from the devices fabricated with halogen‐free organic solvent. Furthermore, the wide bandgap polymer P1 was shown to be able to work well with the narrow bandgap polymer PTB7‐Th in ternary cells, which complemented the absorption of the PTB7‐Th:IEICO‐4F binary cells and increased the *J*_sc_ to 25.18 mA cm^−2^ and the PCE to 12.11%. Interestingly, the energy losses of the devices were ≈0.56 eV, which was among the smallest values reported in literaure. The results demonstrate that FE‐T is an excellent electron‐deficient building block, which can effectively lower polymer HOMO level and optimize the optical property. The results render FE‐T a promising unit to enable polymer semiconductors for efficient NF‐OSCs processable with nonhalogenated solvents.

## Conflict of Interest

The authors declare no conflict of interest.

## Supporting information

Supplementary
