# Supplementary material for: A New Wide Bandgap Donor Polymer for Efficient Nonfullerene Organic Solar Cells with a Large Open‐Circuit Voltage
Source: Adv Sci (Weinh). 2019 Aug 29;6(21):1901773. doi: 10.1002/advs.201901773 (PMC6839623; doi:10.1002/advs.201901773)
Supplement: Supplementary file 1 — Supplementary [file ADVS-6-1901773-s001.pdf]

Copyright WILEY-VCH Verlag GmbH & Co. KGaA, 69469 Weinheim, Germany, 2019.

ADVANCED SCIENCE
Open Access

Supporting Information

for *Adv. Sci.,* DOI: 10.1002/advs.201901773

A New Wide Bandgap Donor Polymer for Efficient Nonfullerene Organic Solar Cells with a Large Open-Circuit Voltage

Yumin Tang, Huiliang Sun, Ziang Wu, Yujie Zhang, Guangye Zhang, Mengyao Su, Xin Zhou, Xia Wu, Weipeng Sun, Xianhe Zhang, Bin Liu, Wei Chen, Qiaogan Liao, Han Young Woo,* and Xugang Guo**

Copyright WILEY-VCH Verlag GmbH & Co. KGaA, 69469 Weinheim, Germany, 2019.

Supporting Information

A New Wide Bandgap Donor Polymer for Efficient Non-Fullerene Organic Solar Cells with A Large Open-Circuit Voltage

Yumin Tang, Huiliang Sun, Ziang Wu, Yujie Zhang, Guangye Zhang, Mengyao Su, Xin Zhou, Xia Wu, Weipeng Sun, Xianhe Zhang, Bin Liu, Wei Chen, Qiaogan Liao, Han Young Woo,* and Xugang Guo**

Y. Tang, Dr. H. Sun, Y. Zhang, M. Su, X. Zhou, X. Wu, W. Sun, X. Zhang, B. Liu, W. Chen, Q. Liao, Prof. X. Guo
Department of Materials Science and Engineering and The Shenzhen Key Laboratory for Printed Organic Electronics, Southern University of Science and Technology (SUSTech), No. 1088, Xueyuan Road, Shenzhen, Guangdong 518055, China
Email: sunhl@sustech.edu.cn (H.S); guoxg@sustech.edu.cn (X.G.)

Z. Wu, Prof. H. Y. Woo
Department of Chemistry, Korea University, Seoul, 136-713, South Korea
Email: hywoo@korea.ac.kr (H.Y.W.)

G. Zhang
eFlexPV Limited (China), Room 228, Block 11, Jin Xiu Da Di, No. 121 Hu Di Pai, Song Yuan Sha Community, Guanhu Street, Longhua District, Shenzhen, China.

Keywords: Wide Bandgap, Donor Polymer, Complementary Absorption, Non-fullerene Organic Solar Cell, Non-Halogenated Solvent

Materials and Instruments

All reagents and chemicals were purchased commercially and used directly without further purification unless otherwise stated. Anhydrous toluene was distilled from Na/benzophenone under argon flow. The donor polymer PTB7-Th (M_w: 180 KDa, PDI: 2.5) and PFN-Br were purchased from 1-MATERIAL INC.. ITIC-Th and IEICO-4F were purchased from VIZUCHEM INC..

Polymer molecular weight was characterized using a high-temperature gel permeation chromatography (GPC, Agilent PL-GPC220) at 150 °C with 1,2,4-trichlorobenzene as the eluent and polystyrenes as the standards. UV-vis absorption spectra of polymer solutions and films and temperature-dependent UV-vis absorption spectra of polymer solutions at various temperatures were collected on the Perkin Elmer Lambda 950 UV/VIS/NIR Spectrometer. Cyclic voltammetry measurements were carried out under argon using a CHI760 Voltammetry Workstation with a N_2-saturated tetra(*n*-butyl)ammonium hexafluorophosphate (Bu_4NPF_6, 0.1 M) solution in acetonitrile (CH_3CN) as the supporting electrolyte. A platinum disk working electrode, a platinum wire counter electrode, and a silver wire reference electrode were employed, and the ferrocene/ferrocenium (Fc/Fc^+) redox couple was used as the reference for all measurements with a scanning rate of 50 mV s^{-1}. The electrochemical onsets were determined at the position the current started to alter from the baseline. The HOMO in electron volts was determined from the onset of the oxidation potential (E_{ox}), according to the following equation: HOMO= $-[4.8+e(E_{ox}-E_{Fc/Fc+})]$. Thermogravimetric analysis (TGA) of polymers was carried out

with a METTLER TOLEDO (TGA 1 STAR^e System) apparatus at a heating ramp of 10 °C min^{-1} under N_2 atmosphere. Differential scanning calorimetry (DSC) curves were recorded on METTLER STAR^e with a heating ramp of 10 ºC min^{-1} in N_2. Room temperature photoluminescence (PL) was recorded by Spectrofluorometer (FS5, Edinburgh instruments). The external quantum efficiency (EQE) spectra of solar cells were recorded by a QE-R3011 measurement system (Enli Technology, Inc.). Atomic force microscopy (AFM) measurements of blend films were conducted using a Dimension Icon Scanning Probe Microscope (Asylum Research, MFP-3D-Stand Alone) in the Pico Center at SUSTech under the tapping mode. Transmission electron microscopy (TEM) specimens were prepared following the identical conditions as the actual devices upon a 30 nm PEDOT:PSS covered substrate. After drying, the substrates were transferred to deionized water and the floated films were transferred onto TEM grids. TEM images were obtained on TEM Tecnai F30 (300 kV) in the Pico Center at SUSTech. Two-dimensional grazing incident wide angle X-ray scattering (2D-GIWAXS) measurements were performed at the PLS-II 9A U-SAXS beam line of Pohang Accelerator Laboratory, Korea.

Synthesis of monomers and polymers

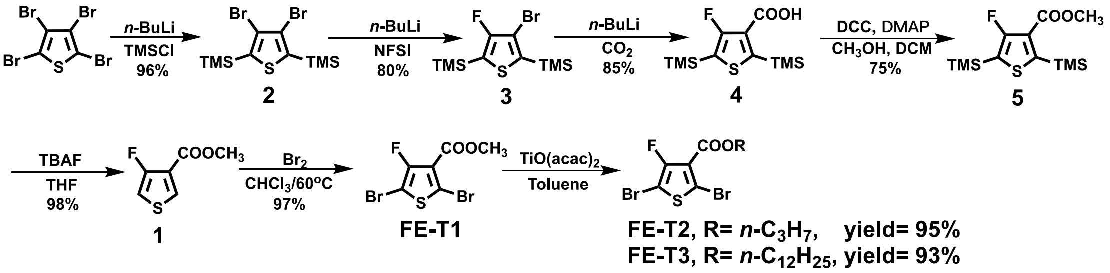

Scheme S1. Synthetic routes to the FE-T monomers.

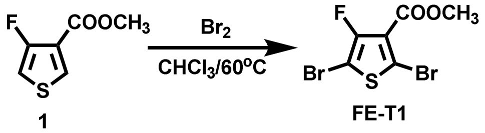

To a solution of compound 1 (1.5 g, 9.37 mmol) in $CHCl_3$ was added Br_2 (2.0 mL, 37.5 mmol, 4.0 eq) and a catalytic amount of $FeCl_3$. The reaction mixture was then heated to 60 °C and stirred at this temperature for 3 h. After cooled to room temperature, the reaction mixture was poured into H_2O and extracted with $CHCl_3$ three times. The combined organic layer was dried over anhydrous Na_2SO_4 and then filtrated. The organic solution was concentrated under reduced pressure and the residue was purified by column chromatography on silica gel using $CHCl_3$ as the eluent to give the monomer FE-T1. ^{1}H NMR (500 MHz, $CDCl_3$) δ (ppm): 3.94 (s, 3H). ^{13}C NMR (126 MHz, $CDCl_3$) δ 159.98, (d, J = 5.0 Hz, C=O) 152.99 (d, J = 273.4 Hz, C-F), 121.92, 117.83, 92.09, 52.39. ^{19}F NMR (376 MHz, $CDCl_3$) δ (ppm): -117.08.

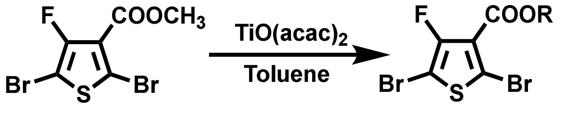

FE-T1

FE-T2, R= $-C_3H_7$
FE-T3, R= $-C_{12}H_{25}$

To a solution of compound FE-T1 (1.0 g, 3.2 mmol) and propan-1-ol or dodecan-1-ol (12.6 mmol, 4 eq) in toluene (50 mL) was added $TiO(acac)_2$ (3.3 g, 12.6 mmol, 4.0 eq). The reaction mixture was then heated to 110 °C and stirred at this temperature for 12 h. After cooled to room temperature, the reaction mixture was poured into H_2O and extracted with $CHCl_3$ three times. The combined organic layer was dried over anhydrous Na_2SO_4 and then filtrated. The organic solution was concentrated under reduced pressure and the residue was purified by column chromatography on silica gel using $CHCl_3$ as the eluent to give the monomer FE-T2 and FE-T3.

FE-T2: ^{1}H NMR (500 MHz, $CDCl_3$) δ (ppm): 4.29 (t, 2H), 1.81-1.92 (m, 2H), 1.04 (t, 3H). ^{13}C NMR (101 MHz, $CDCl_3$) δ (ppm): 159.45 (d, *J*= 5.0 Hz, C=O), 153.07 (d, *J*= 272.7 Hz, C-F), 122.31, 117.45, 92.00, 67.23, 21.91, 10.52. ^{19}F NMR (376 MHz, $CDCl_3$) δ (ppm): -116.96.

FE-T3: ^{1}H NMR (500 MHz, $CDCl_3$) δ (ppm): 4.33 (t, 2H), 1.79-1.72 (m, 2H), 1.79-1.72 (m, 18H), 0.90 (t, 3H). ^{13}C NMR (101 MHz, $CDCl_3$) δ(ppm): 159.47 (d, *J*= 5.0 Hz, C=O), 153.07 (d, *J*= 273.7 Hz, C-F), 122.56, 117.35, 91.75, 65.78, 31.93, 29.65, 29.57, 29.50, 29.36, 29.18, 28.49, 25.90, 22.71, 14.14.^{19}F NMR (376 MHz, $CDCl_3$) δ (ppm): -116.94.

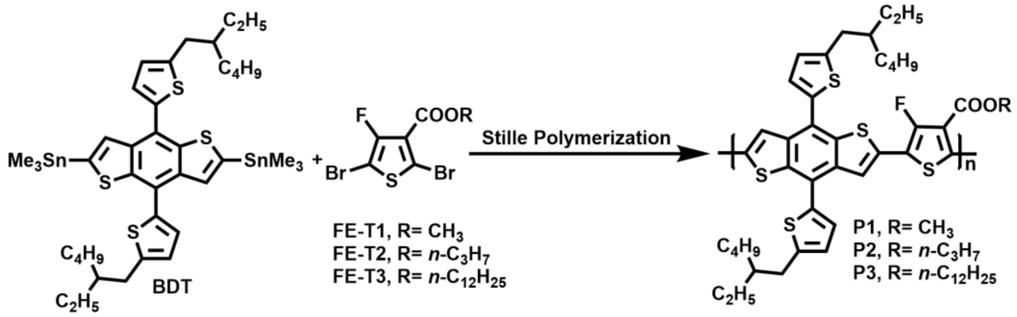

Microwave-assisted polymerization: To a 25 mL reaction tube equipped with a stir bar, a distannylated monomer, BDT (0.5 mmol), dibrominated monomer, FE-T (158 mg, 0.5 mmol), PP2(dba)$_3$ (6.85 mg, 0.0075 mmol), P(*o*-tolyl)$_3$ (18.25 mg, 0.012 mmol), and toluene (10 mL) were added. The tube was purged with argon and sealed. The reaction tube was then set into a microwave reactor and heated at 140 °C for 5 h. After cooling to room temperature, the reaction mixture was dripped into 80 mL methanol containing HCl under vigorous stirring and stirred for 1 h. Then the precipitated solid was filtered through a thimble and subjected to sequential Soxhlet extraction with methanol, hexane, and dichloromethane as solvents to remove impurities and low-molecular-weight fractions. The residue was extracted with chloroform as the final solvent and the chloroform solution was concentrated to ~20 mL and precipitated by dropping into 100 mL methanol. Then, the precipitate was dried *in vacuo* to yield the product polymer as a dark blue solid.

Elemental analysis calcd (%) for P1 (C40H43FO2S5): C= 65.36%, H= 5.90%. Found: C= 65.32%, H= 6.00%.

Elemental analysis calcd (%) for P2 (C42H47FO2S5): C= 66.10%, H= 6.21%. Found: C= 66.00%, H= 6.20%.

Elemental analysis calcd (%) for P3 (C45.6H48F2.8O2S5.6): C= 68.88%, H= 7.37%.

Found: C= 68.80%, H= 7.30%.

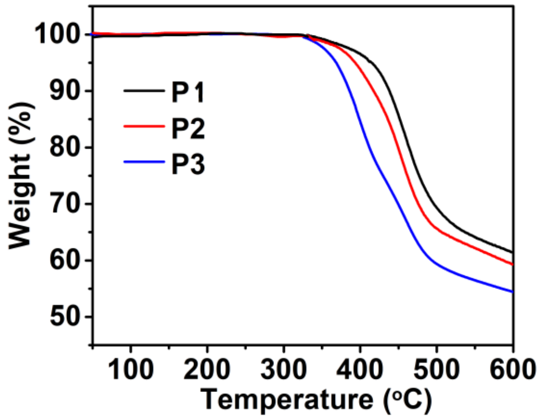

Figure S1. TGA analysis (heating ramp: 10 ºC min^{-1}) of polymers P1, P2 and P3.

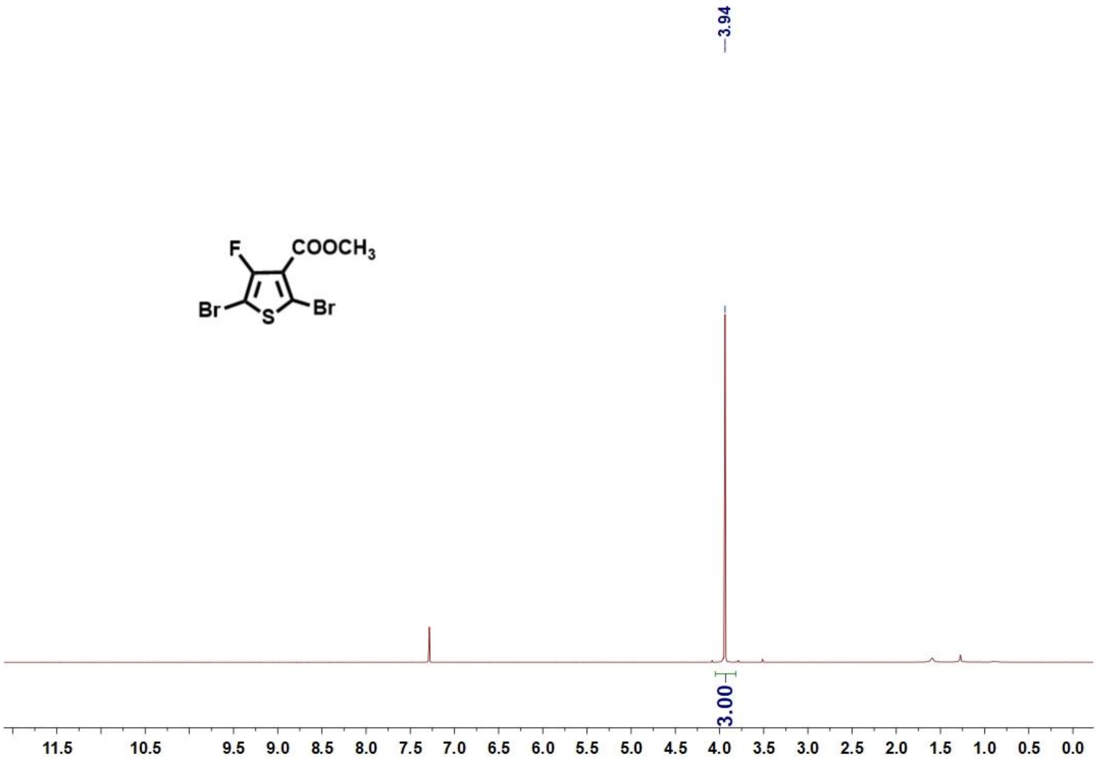

Figure S2. ^{1}H NMR of FE-T1.

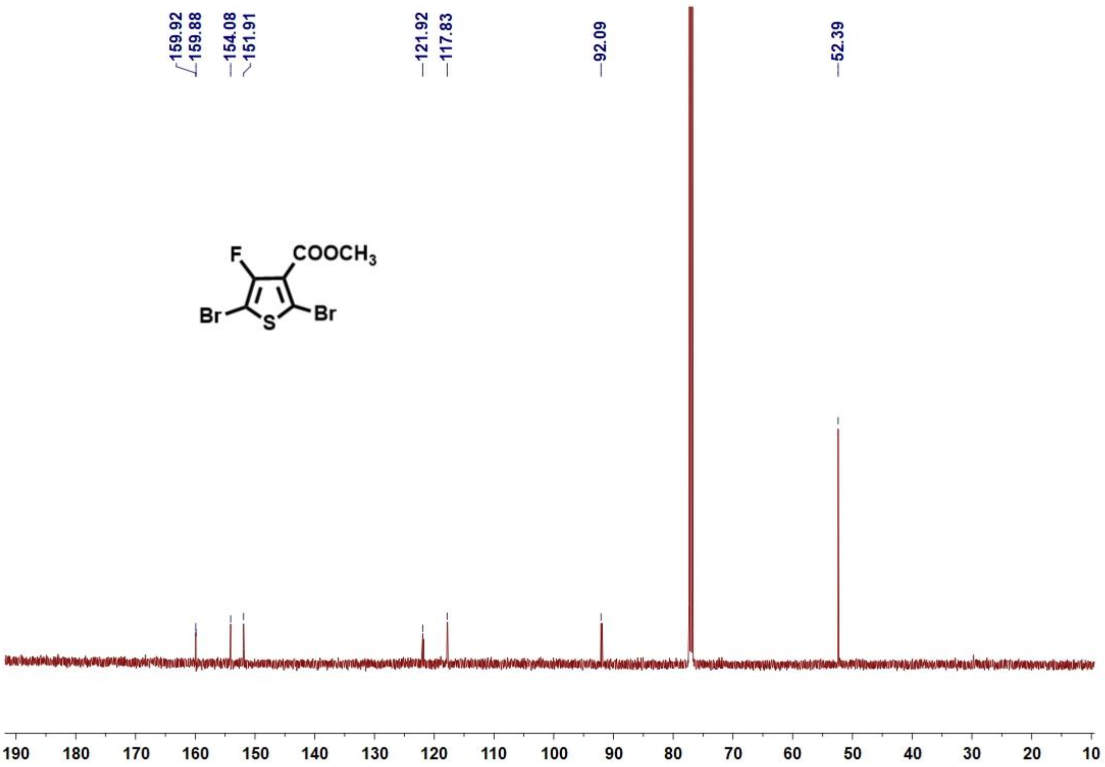

Figure S3. ^{13}C NMR of FE-T1.

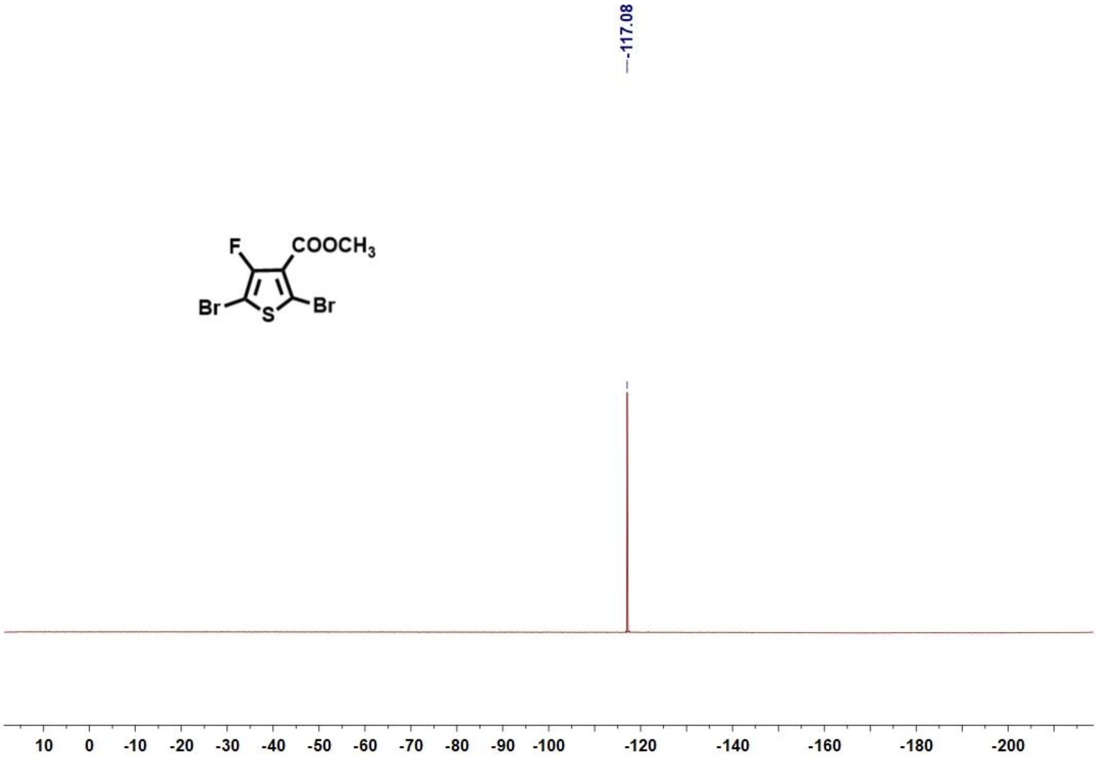

Figure S4. ^{19}F NMR of FE-T1.

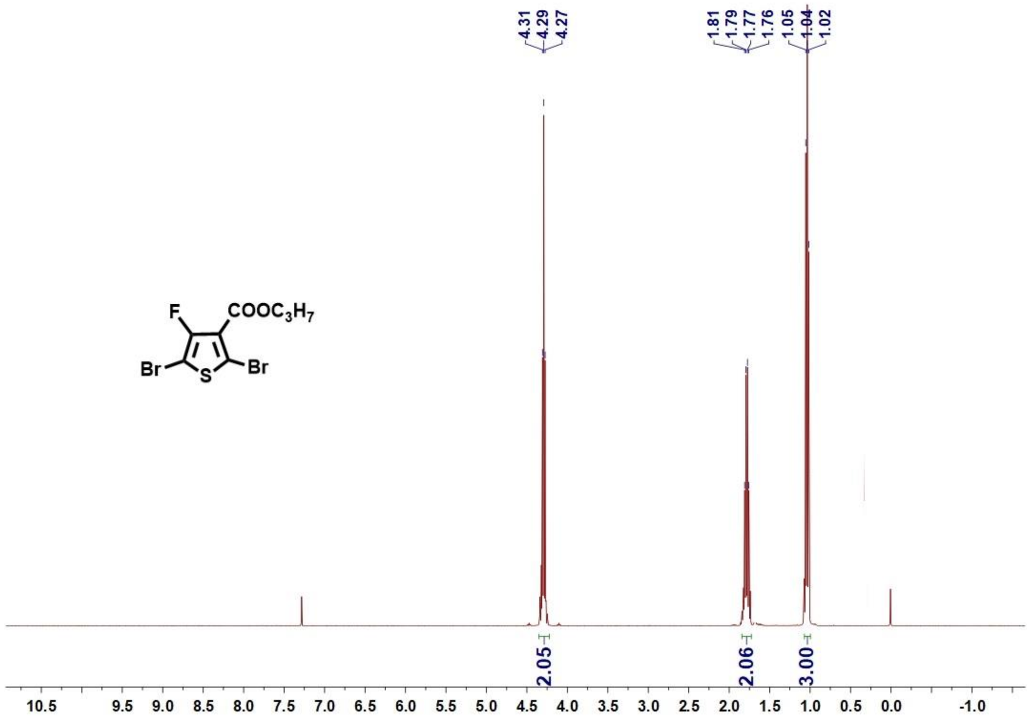

Figure S5. ^{1}H NMR of FE-T2.

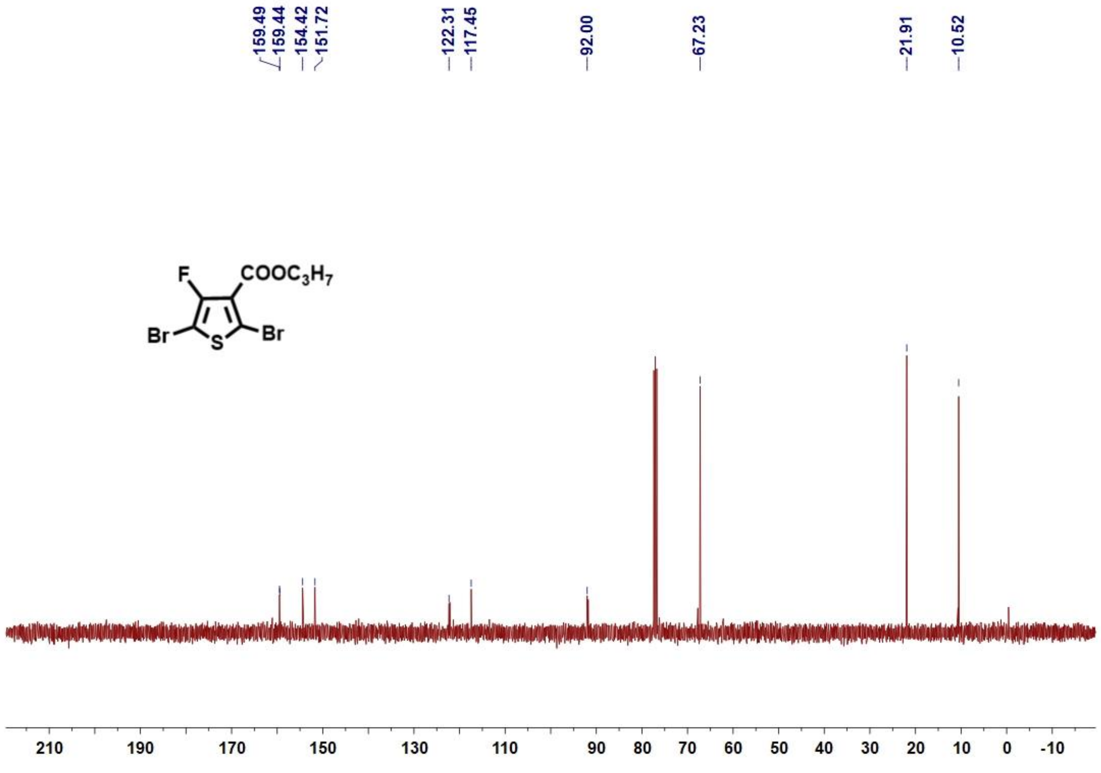

Figure S6. ^{13}C NMR of FE-T2.

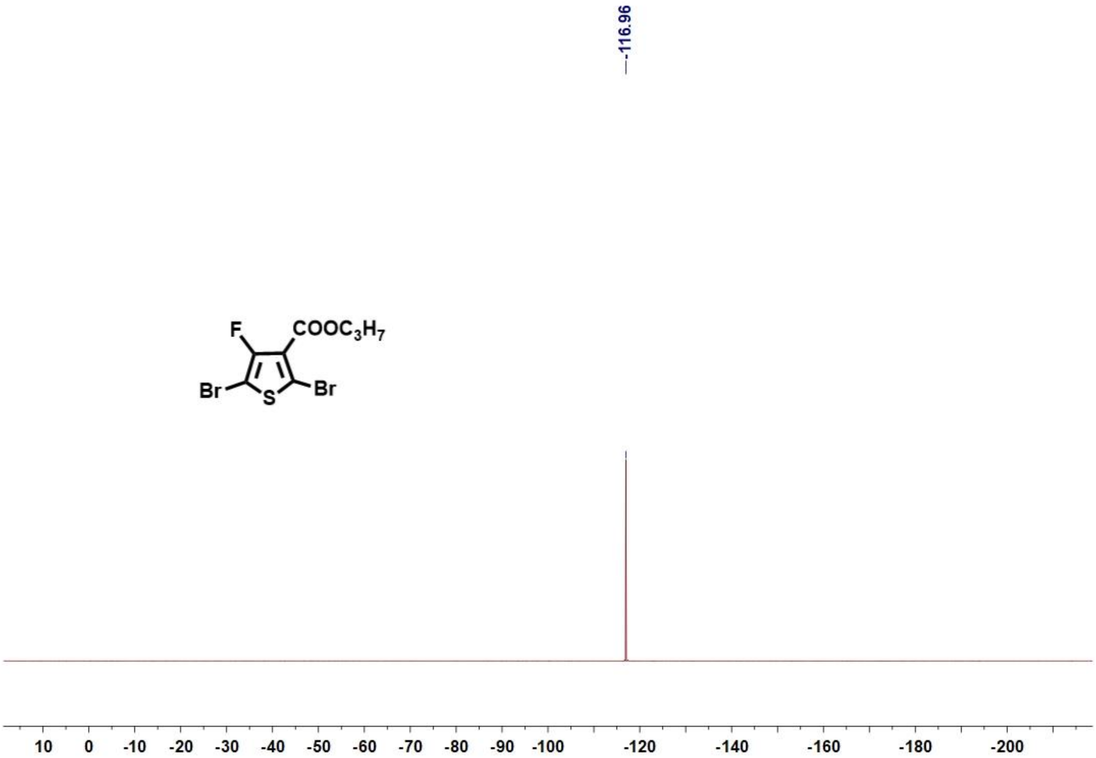

Figure S7. ^{19}F NMR of FE-T2

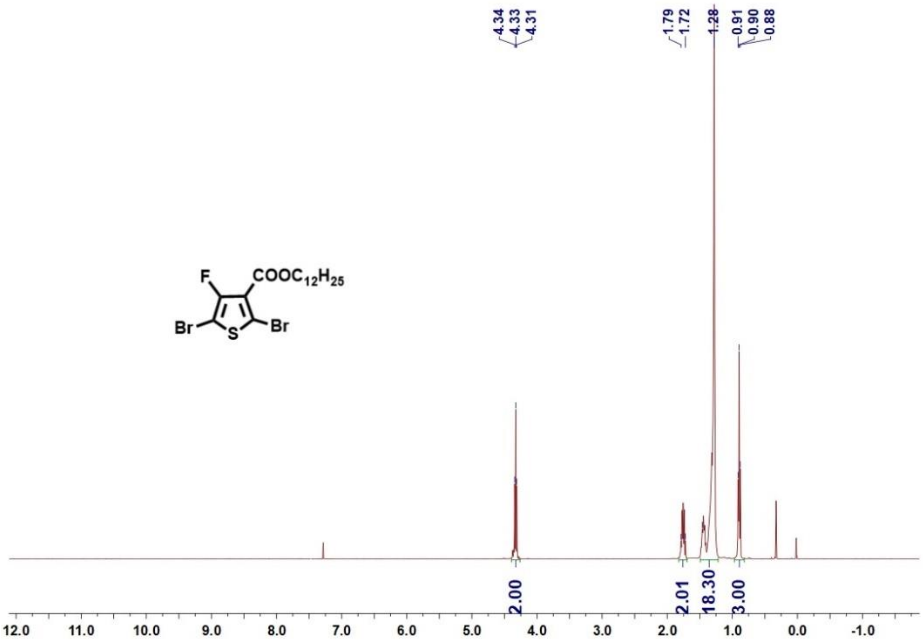

Figure S8. ^{1}H NMR of FE-T3.

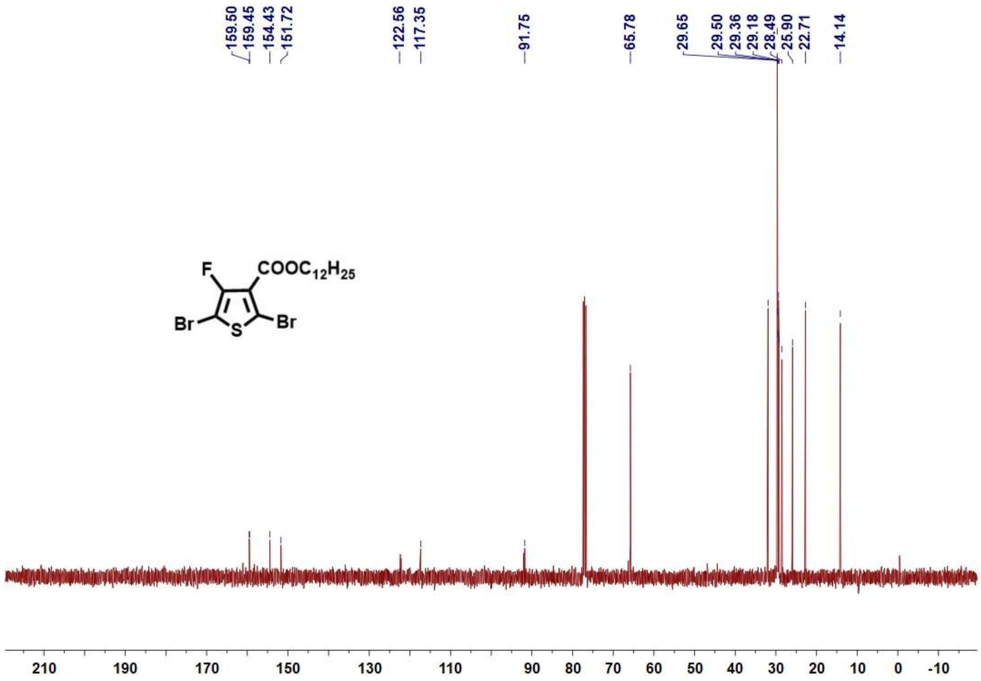

Figure S9. ^{13}C NMR of FE-T3.

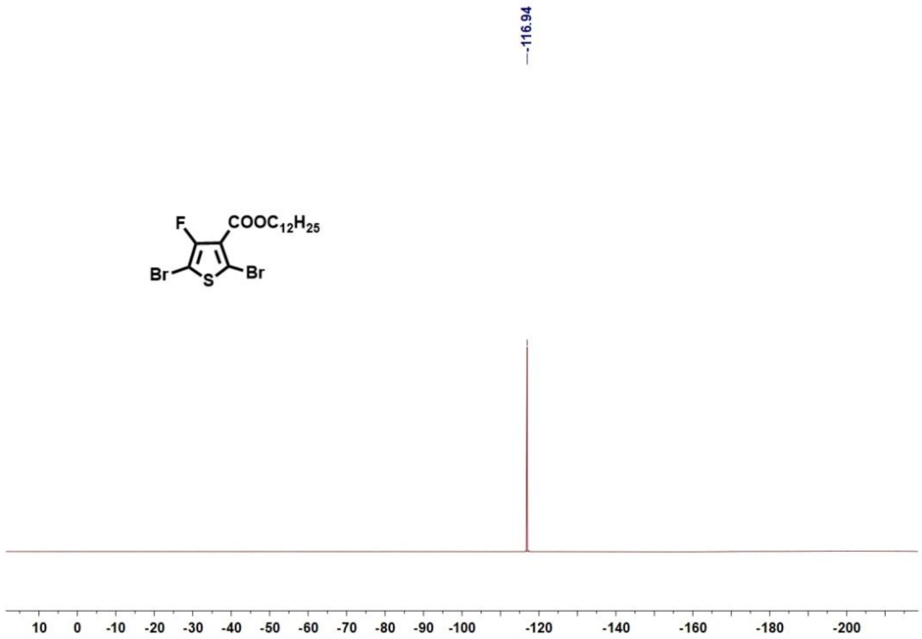

Figure S10. ^{19}F NMR of FE-T3.

Device fabrication and characterization

Binary NF-OSCs Fabrication and Characterization: Conventional binary NF-OSCs with a device structure of glass/ITO/PEDOT:PSS (Clevios P VP AI 4083)/donor:ITIC-Th /Ca or PFN-Br/Al was used for device fabrication and optimization. Pre-patterned ITO-coated glass with a sheet resistance of < 10 Ω/sq was used as the substrate, which was cleaned by sequential sonication in H_2O containing detergent, deionized H_2O, acetone, and isopropanol followed by drying in vacuum oven and then UV-ozone (BZS250GF-TC, HWOTECH, Shenzhen) treatment for 15 min. 40 nm PEDOT:PSS was deposited onto ITO glass substrates via spin-coating as the hole extraction layer, followed by thermal annealing for 10 min at 150 ℃. The active layer with different optimal thicknesses was spin-coated onto PEDOT:PSS from donor:ITIC-Th blend solutions (10 mg/mL for donor) in non-halogenated solvents (heated to solving at 90 ℃) or chlorobenzene solvent (room temperature for solving) under various donor:ITIC-Th weight ratios with or without additives. All the films were thermal annealed at 100 ℃ for 5 min. Then 15nm Ca was thermal evaporated or 10 nm PFN-Br was spin-coated from 0.5 mg/mL methanol solution at 3500 rpm/min and 100 nm Al was sequentially deposited atop the PFN-Br layer via thermal evaporation (ca. 1×10^{-5} Pa) to enable the effective area of 0.045 cm^2 for the solar cells. For solar cell characterization, all current-voltage (*J-V*) characteristics of the devices were measured under simulated AM 1.5G irradiation (100 mW cm-2) using a Xe lamp-based SS-F5-3A Solar Simulator (Enli Technology, Inc.). A Xe lamp equipped with an AM 1.5G filter was used as the light source and the light intensity

was controlled with an NREL-calibrated Si solar cell with a KG-5 filter. The external quantum efficiency (EQE) was measured by a QE-R3011 measurement system (Enli Technology, Inc.).

Ternary NF-OSCs Fabrication and Characterization: Conventional binary NF-OSCs with a device structure of glass/ITO/PEDOT:PSS (Clevios P VP AI 4083) /donor:IEICO-4F/PFN-Br/Al was used for device fabrication and optimization. Pre-patterned ITO-coated glass with a sheet resistance of < 10 Ω/sq was used as the substrate, which was cleaned by sequential sonication in H_2O containing detergent, deionized H_2O, acetone, and isopropanol followed by drying in vacuum oven and then UV-ozone (BZS250GF-TC, HWOTECH, Shenzhen) treatment for 15 min. 40 nm PEDOT:PSS was deposited onto ITO glass substrates via spin-coating as the hole extraction layer, followed by thermal annealing for 10 min at 150 ℃. The active layer with different optimal thicknesses was spin-coated onto PEDOT:PSS from donor:IEICO-4F blend solutions (10 mg/mL for donor) in toluene (heated for 2 h at 80 ℃ for well solving) or CB solvent under donor:IEICO-4F weight ratio of 1.0:1.5 with 2.5 $_{vol}$% DPE additive. While in the ternary NF-OSCs, the PTB7-Th:P1:IEICO-4F would be changed according to different weight ratios between PTB7-Th and P1 with a fixed donors:IEICO-4F weight ratio of 1.0:1.5, at 10 mg/mL for donors in toluene solvent with 2.5 $_{vol}$% DPE additive. All the films were thermal annealed at 90 ℃ for 5 min. Then 10 nm PFN-Br was spin-coated from 0.5 mg/mL methanol solution at 3500 rpm/min and 100 nm Al was sequentially deposited atop the PFN-Br layer via thermal evaporation (ca. 1×10^{-5} Pa) to enable the effective

area of 0.045 cm^2 for the solar cells. For solar cell characterization, all current density-voltage (*J*-*V*) characteristics of the devices were measured under simulated AM 1.5G irradiation (100 mW cm^{-2}) using a Xe lamp-based SS-F5-3A Solar Simulator (Enli Technology, Inc.). A Xe lamp equipped with an AM1.5G filter was used as the light source and the light intensity was controlled with an NREL-calibrated Si solar cell with a KG-5 filter. The external quantum efficiency (EQE) was measured by a QE-R3011 measurement system (Enli Technology, Inc.).

Optimizing process of optoelectronic property

Table S1. Device performance parameters of NF-OSCs with conventional and inverted device structures under condition of D:A= 1:1.5 at 10 mg/mL for donor. Conventional: ITO/PEDOT:PSS/P1:ITIC/Ca/Al and inverted: ITO/ZnO/ P1:ITIC/MoO_3/Ag.

Configuration	V_{oc} [V]	J_{sc} [mA cm^{-2}]	FF [%]	$PCE_{max/avg}$ [%]
conventioal	0.99	16.52	55.41	9.06/8.93
inverted	0.94	16.96	53.06	8.45/8.19

Table S2. Device performance parameters of NF-OSCs device structure: ITO/PEDOT:PSS/active layer/Ca/Al. Various non-fullerene small molecule acceptors were combined with donor P1 to study and select a favorable acceptor. Device fabrication condition: D:A= 1:1.5 at 10 mg/mL for donor.

Acceptor	Solvent	V_{oc} [V]	J_{sc} [mA cm^{-2}]	FF [%]	$PCE_{max/avg}$ [%]
ITIC	Toluene	0.99	16.52	55.41	9.06/8.93
ITIC-Th	Toluene	1.01	16.37	59.34	9.81/9.66
m-ITIC	Toluene	0.98	16.06	50.71	7.98/7.72
IT-M	Toluene	0.97	15.39	51.96	7.60/7.43
o-IDTBR	Toluene	0.95	6.53	39.58	2.45/2.15
IT-4F	Toluene	0.83	18.56	53.14	8.19/8.04
IEICO-4F	Toluene	0.80	4.42	36.69	1.31/1.18
ITIC-Th[a]	Toluene	1.01	17.89	63.05	11.39/11.17
Y6[a]	Toluene	0.91	20.57	58.09	10.87/10.59

[a] Device structure of ITO/PEDOT:PSS/active layer/PFN-Br/Al; 99% toluene +1% ODT (volume) as the processing solvent; D:A= 1:1.5, 10 mg/mL for donor; 100 ℃ thermal-annealing for 5 min.

Table S3. Device performance parameters of NF-OSCs with device structure of ITO/PEDOT:PSS/active layer/Ca/Al. The P1:ITIC-Th blend films were fabricated with a different D:A ratio from 1.5:1 to 1:1.5 (w/w) in toluene solutions at 10 mg/ml for donor.

D:A	V_{oc} [V]	J_{sc} [mA cm^{-2}]	FF [%]	$PCE_{max/avg}$ [%]
1.5:1.0	0.98	15.41	56.33	8.51/8.32
1.0:1.0	1.00	15.52	57.78	8.96/8.74
1.0:1.5	1.01	16.37	59.34	9.81/9.66

Table S4. Device performance parameters of NF-OSCs with device structure of ITO/PEDOT:PSS/active layer/Ca/Al. The P1:ITIC-Th blend films were fabricated with different P1 concentrations from 8 mg/mL to 15 mg/mL in toluene solutions constituted of D:A= 1:1.5.

Concentration [mg/ml]	V_{oc} [V]	J_{sc} [mA cm^{-2}]	FF [%]	$PCE_{max/avg}$ [%]
8.0	1.01	15.70	60.22	9.54/9.17
10.0	1.01	16.37	59.34	9.81/9.66
15.0	1.01	16.93	54.68	9.35/9.11

Table S5. Device performance parameters of NF-OSCs with device structure of ITO/PEDOT:PSS/active layer/Ca/Al. The P1:ITIC-Th blend films were fabricated based on toluene solutions based on D:A= 1:1.5 and 10 mg/mL, followed by various thermal-annealing treatments from 80 ℃ to 120℃ for 5 min.

Temperature [℃]	V_{oc} [V]	J_{sc} [mA cm^{-2}]	FF [%]	PCE$_{max/avg}$ [%]
80	0.99	16.90	59.61	9.97/9.86
100	1.01	17.13	61.85	10.70/10.54
120	1.00	17.08	60.23	10.28/10.19

Table S6. Device performance parameters of NF-OSCs with device structure of ITO/PEDOT:PSS/active layer/Ca/Al. The P1:ITIC-Th blend films were fabricated with various non-halogenated additive solvents in toluene solutions under D:A= 1:1.5 and 10mg/mL for donor and then treated by thermal-annealing at 100℃ for 5 min.

Additive [1 vol%]	V_{oc} [V]	J_{sc} [mA cm^{-2}]	FF [%]	PCE$_{max/avg}$ [%]
DPE	1.01	17.37	59.36	10.41/10.29
ODT	1.01	17.26	60.27	10.50/10.38

Table S7. Device performance parameters of NF-OSCs with device structure of ITO/PEDOT:PSS/active layer/ETL/Al. The P1:ITIC-Th blend films were fabricated based on 99 $_{vol}$% toluene +1 $_{vol}$% ODT blend solutions with D:A= 1:1.5, 10 mg/mL for donor and after that were treated with 100 ℃ thermal-annealing for 5 min, then followed by spin-coating with various electron transporting layers.

ETL	V_{oc} [V]	J_{sc} [mA cm^{-2}]	FF [%]	PCE$_{max/avg}$ [%]
Ca	1.01	17.53	61.85	10.95/10.86
LiF	1.00	16.53	61.64	10.18/10.01
PFN-Br	1.01	17.89	63.05	11.39/11.17
PDINO	1.01	17.18	63.01	10.94/10.83

Table S8. Device performance parameters of NF-OSCs with device structure of ITO/PEDOT:PSS/active layer/ETL/Al. The donor:ITIC-Th blend films were fabricated based on 99 $_{vol}$% solvent +1 $_{vol}$% ODT blend solutions with D:A= 1:1.5, 10 mg/mL for donor and after that were treated with 100 ℃ thermal-annealing for 5 min, then followed by spin-coating with PFN-Br as electron transporting layers.

Materials	Solvent	Thickness (nm)	V_{oc} [V]	J_{sc} [mA cm^{-2}]	FF [%]	PCE$_{max/avg}$ [%]
PTB7-Th:ITIC-Th	CB	105	0.80	15.79	70.02	8.84/8.67
P1:ITIC-Th	CB	115	1.01	18.03	64.82	11.80/11.57
P1:ITIC-Th	*o*-Xylene	100	1.02	16.47	59.17	9.94/9.62
P1:ITIC-Th	TMB	90	1.02	14.78	57.44	8.66/8.35
P1:ITIC-Th [a]	THF	145	1.02	10.18	50.36	5.23/4.93
P1:ITIC-Th [b]	CPME	130	1.01	13.85	55.27	7.73/7.34

[a] 6.0 mg/mL for donor in solution; [b] 7.5 mg/mL for donor in solution, and hot spin-coated at 100℃.

Table S9. Device performance parameters of NF-OSCs with device structure of ITO/PEDOT:PSS/active layer/PFN-Br/Al. The D1:D2:A blend films were fabricated based on 97.5 $_{vol}$% toluene+2.5 $_{vol}$% DPE with various PTB7-Th:P1:IEICO-4F weight ratios while fixed D:A= 1:1.5 and 10 mg/mL for donor, then thermal-annealed at 90 ℃ for 5 min. The values in parentheses meant mass ratios.

Materials	Solvent	V_{oc} [V]	J_{sc} [mA cm^{-2}]	FF [%]	PCE$_{max/avg}$ [%]
PTB7-Th(0.8):P1(0.2)	Toluene	0.73	23.29	64.12	10.8910.72
PTB7-Th(0.7):P1(0.3)	Toluene	0.74	22.14	63.25	10.35/10.21
PTB7-Th(0.6):P1(0.4)	Toluene	0.74	20.53	63.99	9.58/9.40
PTB7-Th(1.0):IEICO-4F(1.5)	CB	0.73	22.89	64.82	10.83/10.56

Table S10. NF-OSC performance parameters based on polymers P2 and P3 with ITIC-Th as acceptor. The donor:ITIC-Th blend films were fabricated based on 99 $_{vol}$% solvent +1 $_{vol}$% ODT blend solutions with D:A= 1:1.5, 10 mg/mL for donor and after that were treated with 100 ℃ thermal-annealing for 5 min, then followed by spin-coating with PFN-Br as electron transporting layers.

Materials	Thickness (nm)	PCE$_{max/avg}$ (%)	V_{oc} (V)	J_{sc}^{a} (mA cm^{-2})	J_{sc}^{b} (mA cm^{-2})	FF (%)	$E_g^{onset\ c}$ (eV)	E_{loss} (eV)
P2:ITIC-Th	85	2.35/2.11	0.91	8.68	8.63	29.66	1.60	0.69
P3:ITIC-Th	100	3.35/3.18	1.00	9.62	9.60	34.65	1.59	0.59

[a] Obtained from *J-V* measurements; [b] Integrated from EQE; [c] Calculated from EQE spectrum.

Optoelectronic performance of binary NF-OSCs

To investigate the molecular conformations and electronic structure, density functional theory (DFT) calculations were performed at □B97X-D/6-31G(d,p) level of theory using Gaussian 16 A.03 program. As shown in **Figure S11(a)**, compared with the monomer of PTB7-Th, both of the two possible regioselective linkage of repeated unit of P1 was found in more planar structure, which could be attributed to intramolecular non-covalent O···S, F···S and F···O interaction and smaller steric hindrance after releasing the thiophene unit connecting to the backbone. Elimination of the thiophene can also effectively lower down the energy level of frontier molecular orbital, which would contribute to larger open-circuit voltage. Theoretical

calculation based on trimers suggests that the HOMO energy level of P1 was lower than that of PTB7-Th by 0.23 eV. Furthermore, as presented in **Figure S11(b)**, simulation of optical spectrum using TD-DFT at tuned-ωB97X-D/6-31G(d,p)//ωB97X-D/6-31G(d,p) theoretical level indicated an obviously hypsochromic shift of 75 nm in the absorption spectrum of P1, which would be ascribed to the elimination of electron-donating thiophene and more terse conjugated unit.

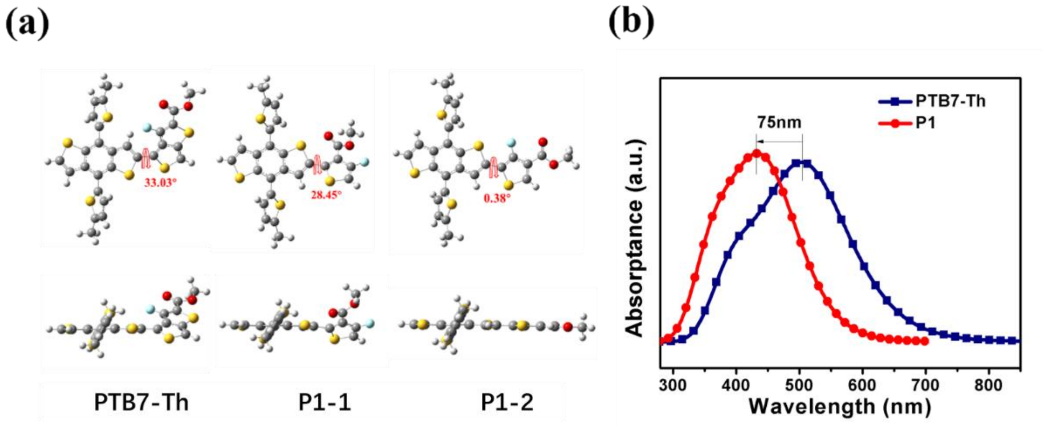

Figure S11. (a) Optimized molecular structure and its side view, at DFT/ωB97X-D/6-31G(d,p) theoretical level; (b) DFT-simulated optical spectrum of trimers of PTB7-Th and P1 at tuned-ωB97X-D/6-31G(d,p)//ωB97X-D/6-31G(d,p) theoretical level, where ω are 0.0883 and 0.0991 respectively via ionization potential tuning procedure.

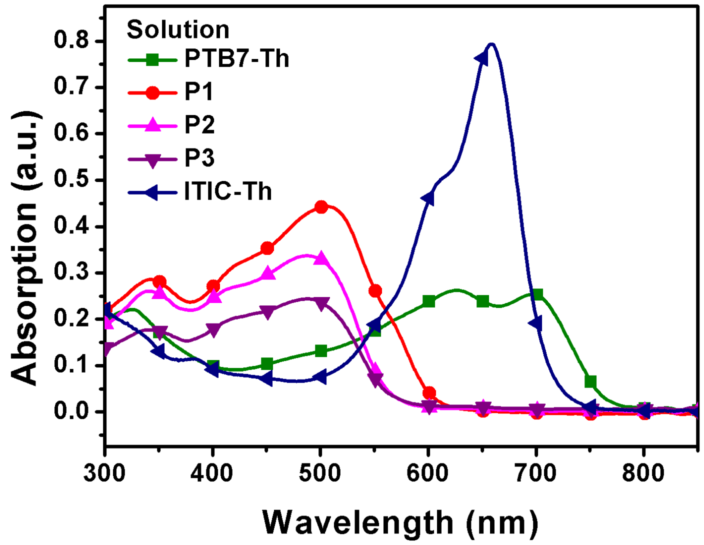

Figure S12. Absorption spectra of PTB7-Th, P1-P3 and ITIC-Th in toluene solvent at 0.01 mg/ml.

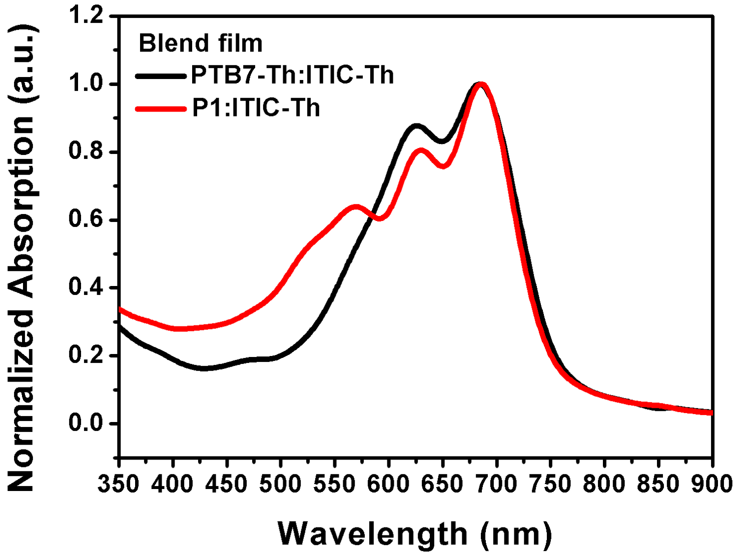

Figure S13. Absorption spectra of PTB7-Th:ITIC-Th and P1:ITIC-Th in film state.

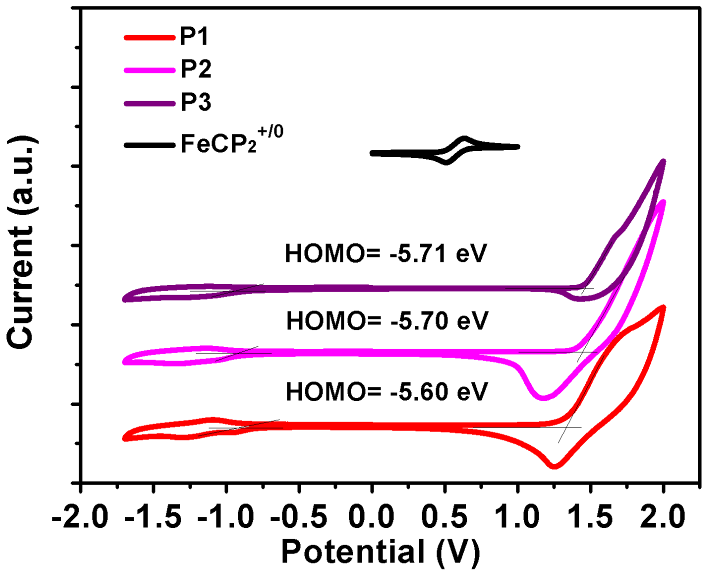

Figure S14. Cyclic voltammograms of P1, P2 and P3 with $FeCP_2^{+/0}$ as the standard.

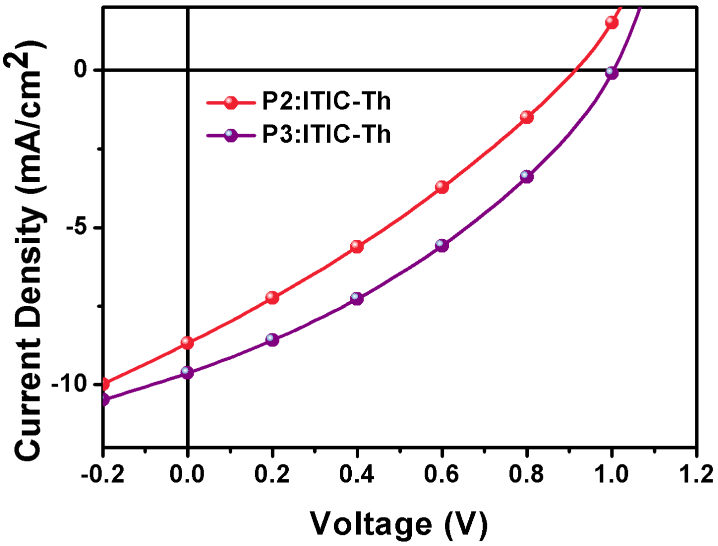

Figure S15. The *J-V* curves of the conventional devices based on P2:ITIC-Th and P3:ITIC-Th blends in toluene solution under illumination (simulated AM 1.5G) at 100 mW/cm^2.

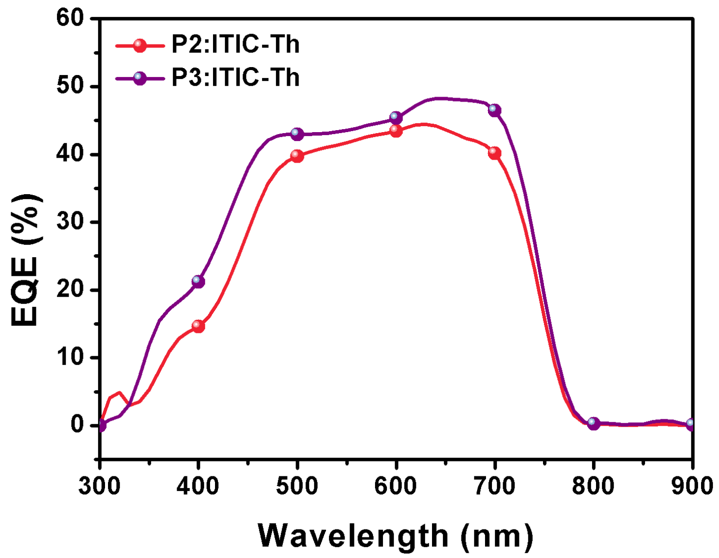

Figure S16. EQE curves of the conventional devices based on P2:ITIC-Th and P3:ITIC-Th blends in toluene solution.

Morphology characterization

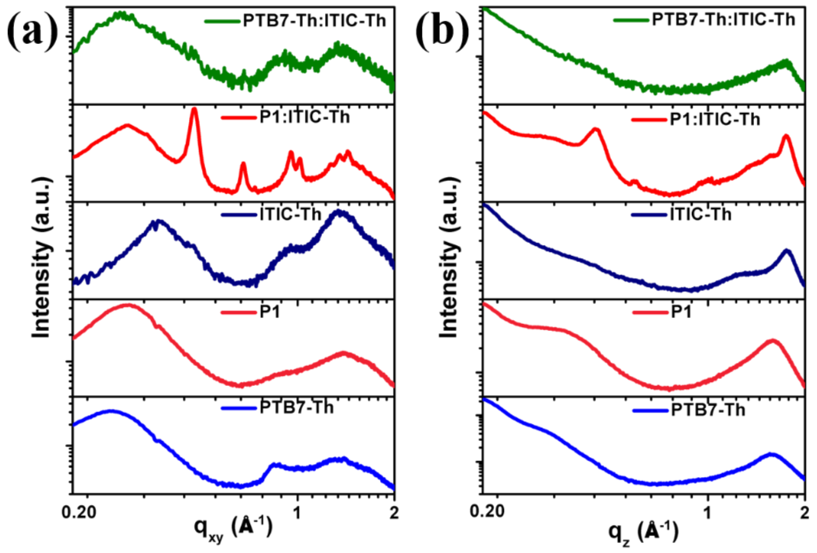

Figure S17. (a) In-Plane and (b) Out-of-Plane one dimensional integrated scattering profiles of the neat film of PTB7-Th, P1 and ITIC-Th, and blend film of PTB7-Th:ITIC-Th and P1:ITIC-Th.

Table S11. Crystallographic Parameters of the neat films and blend films based on PTB7-Th, P1 and ITIC-Th.

Polymer	Crystallographic Parameters		Pristine	Blend with ITIC-Th
PTB7-Th	q_{xy} profile (100)	q (Å^{-1})	0.27	0.28
		d-spacing (Å)	23.26	22.43
		coherence length (nm)	8.97	5.23
	q_z profile (010) π–π stacking	q (Å^{-1})	1.55	1.75
		d-spacing (Å)	4.05	3.59
		coherence length (nm)	1.36	1.46
P1	q_{xy} profile (100)	q (Å^{-1})	0.30	0.29
		d-spacing (Å)	20.93	21.65
		coherence length (nm)	6.97	5.23
	q_z profile (010) π–π stacking	q (Å^{-1})	1.60	1.75
		d-spacing (Å)	3.92	3.59
		coherence length (nm)	1.21	5.709

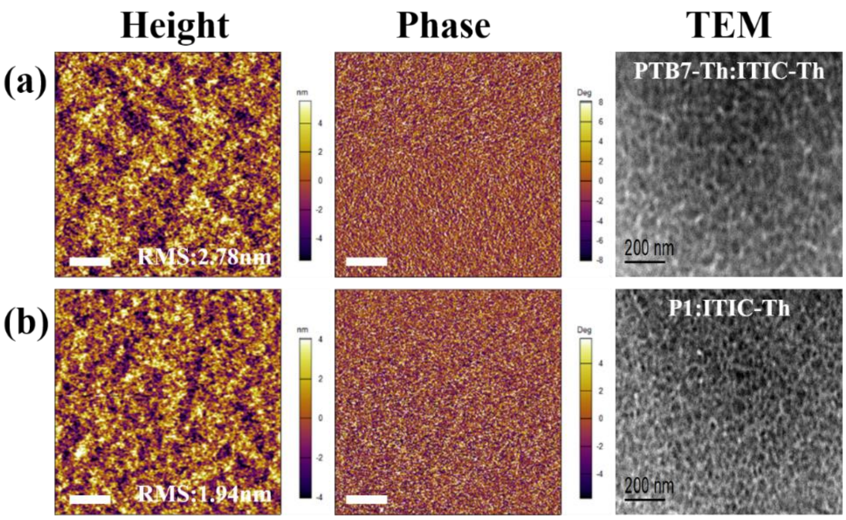

Figure S18. AFM height, phase images and TEM images of (a) PTB7-Th:ITIC-Th blend film and (b) P1:ITIC-Th blend film fabricated from solvent chlorobenzene; Scale bar in AFM images: 1.0 um.

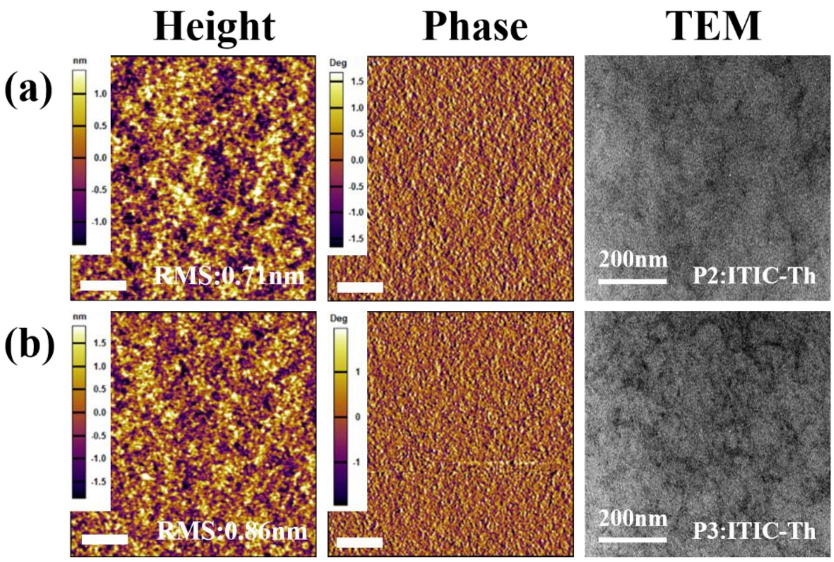

Figure S19. AFM height, phase images and TEM images of (a) P2:ITIC-Th blend film and (b) P3:ITIC-Th blend film; Scale bar in AFM images: 1.0 um.

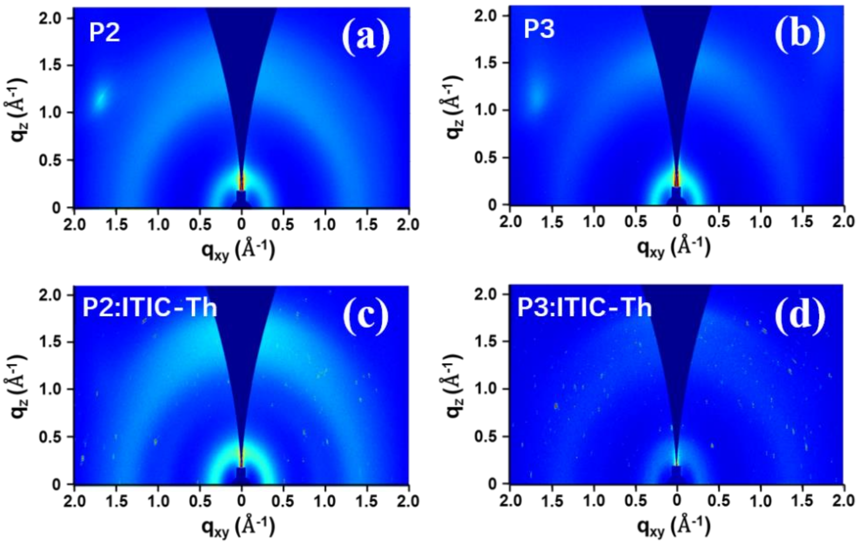

Figure S20. 2D GIWAXS images of neat films based on (a) P2 and (b) P3, and blend films fabricated from (c) P2:ITIC-Th and (d) P3:ITIC-Th.

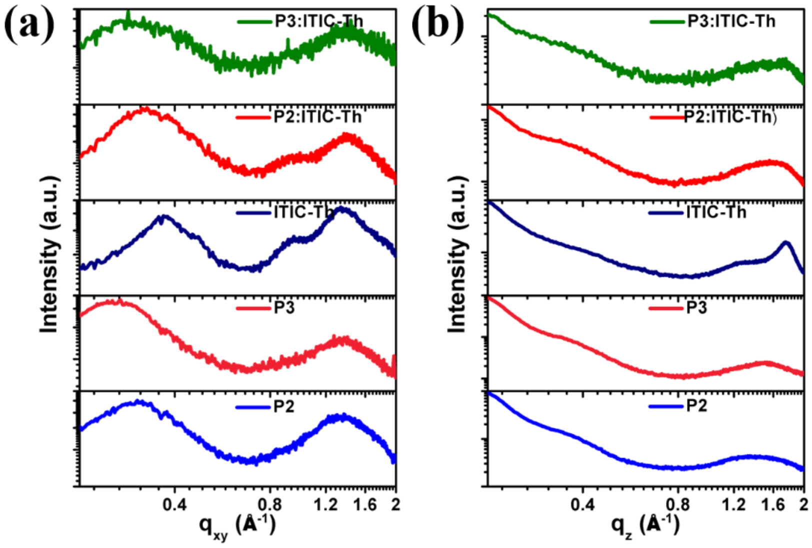

Figure S21. (a) In-Plane and (b) Out-of-Plane one dimensional integrated scattering profiles of the neat film of P2, P3 and ITIC-Th, and blend film of P2:ITIC-Th and P3:ITIC-Th.

SCLC Mobility Measurement

Hole-only and electron-only mobilities were measured using the space-charge-limited current (SCLC) method. The structure of ITO/PEDOT:PSS/active aayer/MoO_3/Ag was used for hole-only devices and the

structure of ITO/ZnO/active layer/PFN-Br/Al was used for electron-only devices, respectively. The SCLC mobilities were calculated by the MOTT-Gurney equation:

$$J = \frac{9}{8}\varepsilon_0\varepsilon_r\mu\frac{V^2}{d^3}$$

Where J was the current density, ε_r was the relative dielectric constant of active layer material, usually 2-4 for organic semiconductors, herein we used a relative dielectric constant of 3 for polymer. ε_0 was the permittivity of empty space, μ was the mobility of hole or electron and d was the active layer thickness, V was the internal voltage in the device, and $V = V_{appl}-V_{bi}$, where V_{appl} was the voltage applied to the device, and V_{bi} was the built-in voltage resulting from the relative work function difference between the two electrodes (in the hole-only and the electron-only devices, the V_{bi} values could be neglected).

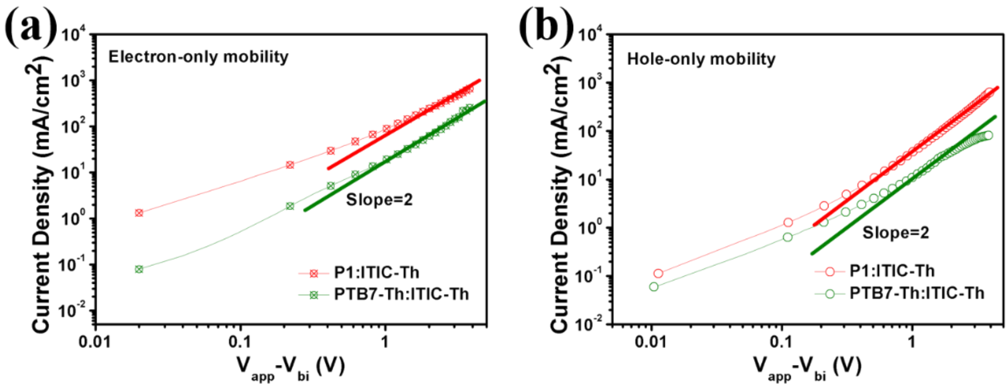

Figure S22. *J-V* plots of the (a) electron-only and (b) hole-only devices based on PTB7-Th:ITIC-Th and P1:ITIC-Th blends fabricated from toluene solution under dark condition.

Table S12. The charge carrier transport mobility parameters of the electron-only and hole-only devices constructed from PTB7-Th:ITIC-Th and P1:ITIC-Th.

Materials	$\mu_{e,sclc}$ [cm^2/v/s]	$\mu_{h,sclc}$ [cm^2/v/s]	μ_e/μ_h
PTB7-Th:ITIC-Th	2.0×10^{-4}	1.8×10^{-4}	1.11
P1:ITIC-Th	3.2×10^{-4}	4.8×10^{-4}	0.67

Photoluminescence characterization

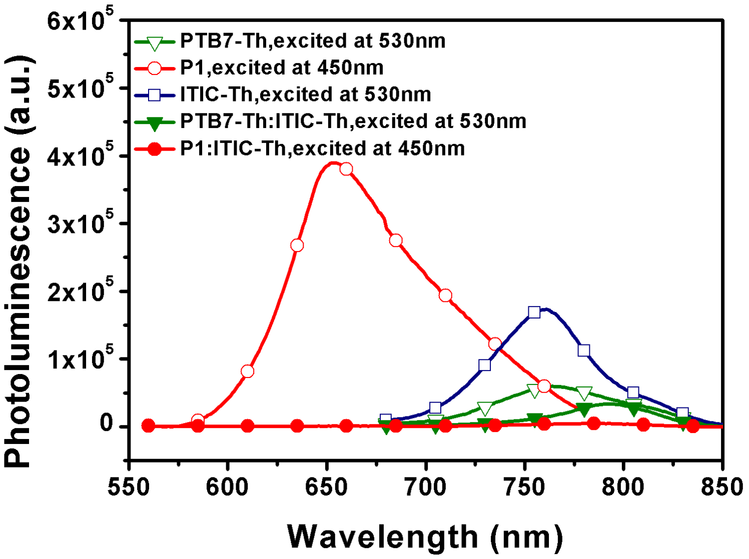

Figure S23. Photoluminescence spectra of the neat films of PTB7-Th, P1 and ITIC-Th as well as the blend films based on PTB7-Th:ITIC-Th and P1:ITIC-Th in the binary NF-OSCs; The excited energy were also shown respectively.

Optoelectronic property of ternary NF-OSCs

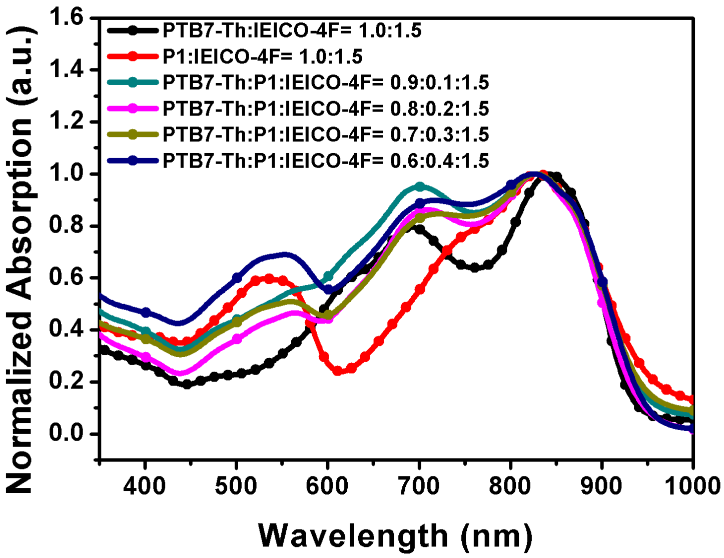

Figure S24. Absorption spectra of binary blend films based on PTB7-Th:IEICO-4F and P1:IEICO-4F and ternary blend films with different weight ratios from 0.1 to 0.4 of P1 in PTB7-Th:IEICO-4F.

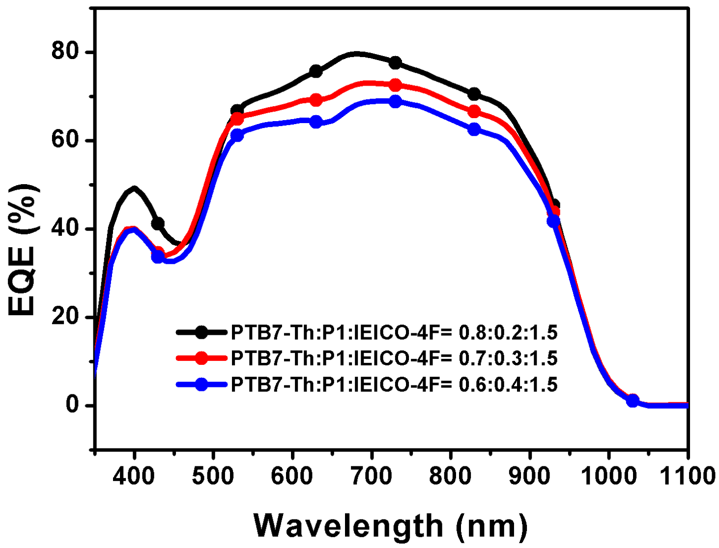

Figure S25. EQE curves of the ternary NF-OSCs based on PTB7-Th:P1:IEICO-4F in toluene solution with various weight ratios of P1 under illumination (simulated AM 1.5G) at 100 mW/cm^2.

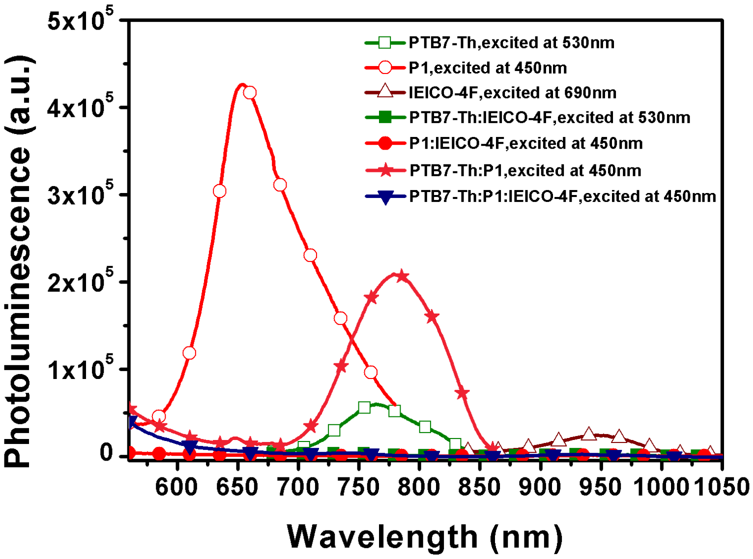

Figure S26. Photoluminescence spectra of the neat films of PTB7-Th, P1 and IEICO-4F as well as the blend films based on PTB7-Th:IEICO-4F, P1:IEICO-4F, PTB7-Th:P1 and PTB7-Th:P1:IEICO-4F in the ternary NF-OSCs; The excited energies were also shown respectively.

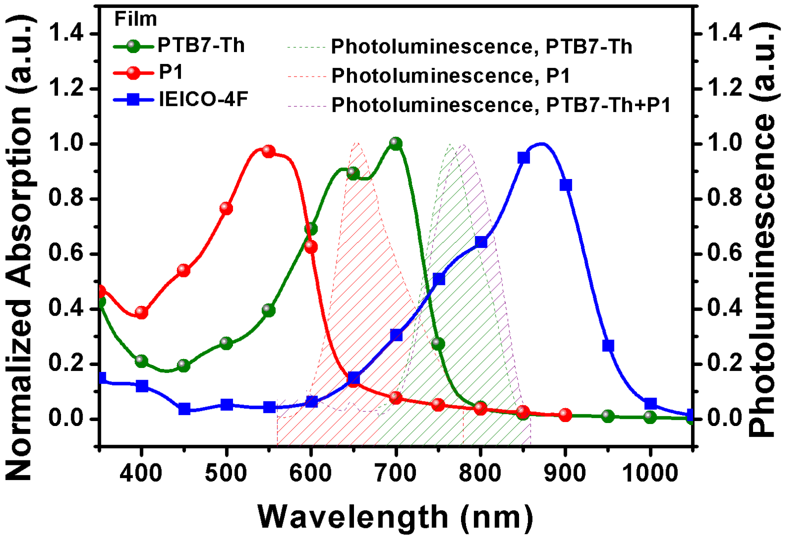

Figure S27. Absorption and photoluminescence spectra of the neat films of PTB7-Th, P1 and IEICO-4F for illustration of the potential non-radiative Förster resonance energy transfer (FRET) in the blend system.

Table S13. Photo-electronic parameters of some advanced works based on various wide-bandgap donors combining with ITIC-Th as the acceptor.

Work	Donor, E_{bg}^{opt}	Acceptor	Solvent	Binary(B)/ Ternary(T)	V_{oc} [V]	J_{sc} [mA/cm^2]	FF [%]	PCE [%]	E_{loss} [eV]	Reference
(1)	PDBT-T1, 1.85eV	ITIC-Th	CF	B	0.88	16.24	67.10	9.60		[1]
(2)	PBnDT-FTAZ, 2.00eV	ITIC-Th	CF	B	0.92	15.84	61.26	8.88		[2]
(3)	PTFB-O, 2.0eV	ITIC-Th	CB	B	0.92	17.10	67.00	10.50		[3]
(4)	PBDB-T, 1.83eV	ITIC-Th	CB	B	0.80	14.20	57.00	6.56		[4]
(5)	PBDT-ODZ, 2.07eV	ITIC-Th	CB	B	1.06	17.02	68.10	12.34	0.50	[5]
(6)	PBT1-EH, 1.84eV	ITIC-Th	CF	B	0.97	15.52	68.10	10.30		[6]
(7)	P1, 1.95eV	ITIC-Th	Toluene	B	1.01	17.89	63.05	11.39	0.57	This Work

Table S14. Photo-electronic parameters of some advanced BHJ-NF-OSCs based on various wide-bandgap donors processed with non-halogenated solvents.

Work	Donor, E_{bg}^{opt}	Acceptor	Solvent	Binary(B)/ Ternary(T)	V_{oc} [V]	J_{sc} [mA/cm^2]	FF [%]	PCE [%]	E_{loss} [eV]	Reference
(1)	PBnDT-FTAZ$_{NO}$, 2.00eV	IT-M	Toluene	B	0.87	15.44	52.50	7.06		[7]
(2)	PBnDT-FTA, 2.00eV	IT-M	LM/2-Me THF	B	0.96	18.30	70.00	12.20		[8]
(3)	PBnDT-FTA(2.00eV), PBDB-T(1.83eV)	IT-M	Toluene	T	0.95	18.10	73.60	12.70		[9]
(4)	PBDT-S-2TC	ITIC	TMB	B	0.92	17.32	60.05	9.55		[10]
(5)	PBDTS-TDZ, 2.07eV	ITIC	*o*-Xylene	B	1.10	17.78	65.40	12.80	0.48	[11]

(6)	PM6, 1.89eV	IT-4F	Xylene/n-Butyl Alcohol	B	0.84	20.50	75.00	12.9		[12]
(7)	P1(1.95eV), PTB7-Th(1.56eV)	IEICO-4F	Toluene	T	0.74	25.18	65.19	12.11	0.56	This Work

References

[1] Y. Lin, F. Zhao, Q. He, L. Huo, Y. Wu, T. C. Parker, W. Ma, Y. Sun, C. Wang, D. Zhu, A. J. Heeger, S. R. Marder, X. Zhan, *J. Am. Chem. Soc.* **2016**, *138*, 4955.

[2] F. Zhao, S. Dai, Y. Wu, Q. Zhang, J. Wang, L. Jiang, Q. Ling, Z. Wei, W. Ma, W. You, C. Wang, X. Zhan, *Adv. Mater.* **2017**, *29*, 1700144.

[3] Z. Li, K. Jiang, G. Yang, J. Y. Lai, T. Ma, J. Zhao, W. Ma, H. Yan, *Nat. Commun.* **2016**, *7*, 13094.

[4] J. Mai, Y. Xiao, G. Zhou, J. Wang, J. Zhu, N. Zhao, X. Zhan, X. Lu, *Adv. Mater.* **2018**, *30*, 1802888.

[5] X. Xu, Z. Li, Z. Bi, T. Yu, W. Ma, K. Feng, Y. Li, Q. Peng, *Adv. Mater.* **2018**, *30*, 1800737.

[6] T. Liu, X. Pan, X. Meng, Y. Liu, D. Wei, W. Ma, L. Huo, X. Sun, T. H. Lee, M. Huang, H. Choi, J. Y. Kim, W. C. Choy, Y. Sun, *Adv. Mater.* **2017**, *29*, 1604251.

[7] Z. Chen, L. Yan, J. J. Rech, J. Hu, Q. Zhang, W. You, *ACS Appl. Polym. Mater.* **2019**, *1*, 804.

[8] L. Ye, Y. Xiong, Z. Chen, Q. Zhang, Z. Fei, R. Henry, M. Heeney, B. T. O'Connor, W. You, H. Ade, *Adv. Mater.* **2019**, *31*, 1808153.

[9] H. Hu, L. Ye, M. Ghasemi, N. Balar, J. J. Rech, S. J. Stuard, W. You, B. T. O'Connor, H. Ade, *Adv. Mater.* **2019**, *31*, 1808279.

[10] Y. An, X. Liao, L. Chen, J. Yin, Q. Ai, Q. Xie, B. Huang, F. Liu, A. K. Y. Jen, Y. Chen, *Adv. Funct. Mater.* **2018**, *28*, 1706517.

[11] X. Xu, T. Yu, Z. Bi, W. Ma, Y. Li, Q. Peng, *Adv. Mater.* **2018**, *30*, 1703973.

[12] S. Dong, K. Zhang, B. Xie, J. Xiao, H.-L. Yip, H. Yan, F. Huang, Y. Cao, *Adv. Energy Mater.* **2019**, *9*, 1802832.
